# Comprehensive analysis of lncRNAs modified by m^6^A methylation in sheep skin

**DOI:** 10.5713/ab.24.0039

**Published:** 2024-05-07

**Authors:** Jinzhu Meng, Jianping Li, Yuanyuan Zhao

**Affiliations:** 1 Guizhou Provincial Key Laboratory for Biodiversity Conservation and Utilization in the Fanjing Mountain Region, Tongren University, Tongren, Guizhou 554300, China; 2 College of Veterinary Medicine, Hunan Agricultural University, Changsha, Hunan 410128, China; 3 College of Animal Science and Technology, Jilin Agricultural Science and Technology University, Jilin, Jilin 132000, China

**Keywords:** lncRNA, m^6^A Methylation, Skin Pigmentation, Sheep

## Abstract

**Objective:**

N6-methyladenosine (m^6^A) is the most prevalent methylation of mRNA and plays crucial roles in various physiological processes, including pigmentation. Yet, the regulatory mechanisms, including long noncoding RNAs (lncRNAs) m^6^A methylation contributing to pigmentation in sheep skin remains unclear. The purpose of this study was to identify potential lncRNAs and the m^6^A methylation of lncRNAs associated with pigmentation.

**Methods:**

RNA-seq and MeRIP-seq were performed to study the expression of lncRNAs and the m^6^A methylation of lncRNAs in black and white sheep skin. Furthermore, quantitative real-time polymerase chain reaction (qRT-PCR) was used to verify the consistency with the RNA-seq and MeRIP-seq data.

**Results:**

We identified 168 differentially expressed lncRNAs between the two sheep skin colors. The differentially expressed lncRNAs enriched in the pathway of ECM-receptor interaction, Rap1 signaling pathway, and Non-homologous end-joining may play essential roles in pigmentation. We identified 577 m^6^A peaks and 617 m^6^A peaks in black and white sheep skin, respectively, among which 20 m^6^A peaks showed significant differences. The enriched motif in sheep skin was “GGACU”, which aligned with the consensus motif “RRACH” (R = A or G, H = A, C or U). Differently methylated lncRNAs enriched in PI3K-Akt signaling pathway and Wnt signaling pathway might participate in skin pigmentation. ENSOARG00020015168 was the unique lncRNA with high expression and methylation (Hyper-Up) in black sheep shin. A lncRNA-mRNA network was constructed, with pigmentation-related genes, such as *PSEN2*, *CCND3*, *COL2A1*, and *ERCC3*.

**Conclusion:**

The m^6^A modifications of lncRNAs in black and white colored sheep skin were analyzed comprehensively, providing new candidates for the regulation of pigmentation.

## INTRODUCTION

Coat color variation is a sign of domestication and a typical trait of some animal breeds, which not only has high significance as ornamental or economic value but also is closely related to animal health. Thus, revealing the genetic basis of coat color is important for molecular breeding in fur animals. Coat color is affected by the melanin production, eumelanin/pheomelanin ratio and the pigmentation intensity in melanocytes, and the distribution of melanin along hairs. More than 150 genes have been discovered in this regard, which play important roles in melanocyte migration, differentiation, melanin production, transport, and regulation [1]. Several examples are found in tyrosinase (TYR) families that catalyze dopaquinone and eumelanin synthesis [2]. Microphthalmia-associated transcription factor (MITF) plays an important role in melanogenesis, melanin transport, signal transduction pathways, as well as melanocyte migration and differentiation [3]. In addition, alpha-melanocyte stimulating hormone (α-MSH), stem cell factor (SCF), and endothelin 1 (ET-1) serve as paracrine factors to activate specific internal signaling pathways in melanogenesis, which include cAMP/PKA, MAPK, PI3K/Akt, SCF/c-Kit, and Wnt/β-catenin signaling pathways [4–6].

LncRNAs are noncoding transcripts that are transcribed by RNA polymerase II, >200 bp in length, and possess a wide range of functions by cis-or trans-action, including melanogenesis [7,8]. The lncRNA expression profiles of mouse skin [9], mouse melanocytes [10], goat skin and melanocytes [1, 11], sheep skin [12], and pig skin [13] have been obtained. Some pigment-related lncRNAs have also been validated. For example, H19 stimulate the production of melanin in melanocytes through promoting the secretion of α-MSH in keratinocytes and by H19-derived miR-675 directly target MITF [14,15]. In addition, SPRIGHTLY and TCONS_000 49140 may regulate cell proliferation in primary human melanocytes [9,16]. ENST00000606533-miR-1291-TYR promotes melanogenesis [17], whereas SRA, UCA1, and TUG1 may negatively regulate melanogenesis in melanocytes [18–20]. Meanwhile, α-MSH stimulation and UVB irradiation affect the expression of lncRNAs in human melanocytes [17,21].

Among known epigenetic mechanisms, DNA methyla tion, non-coding RNA, histone modification, and chromatin reMeRIP-seqmodeling have been proved to regulate genes involved in melanogenesis [7]. However, few studies have focused on RNA methylation affecting melanogenesis. N6-methyladenosine (m^6^A) is the most universal mRNA modification present across various species [22–25]. The m^6^A methylation is recognized, catalyzed, and removed by reader proteins, methyltransferases, and demethylases, respectively [26,27]. The m^6^A modifications may play important roles in metabolism, growth, development, immune responses, reproduction, and other physiological processes [28–30]. We previously identified pigment-associated m^6^A modifications of mRNA in sheep skin [31]. However, the role of lncRNAs m^6^A methylation in sheep skin has not been reported.

Therefore, in this work, we performed RNA sequencing (RNA-seq) and methylated-RNA immunoprecipitation and sequencing (MeRIP-seq) of black and white sheep skin to investigate pigment-related lncRNAs and m^6^A methylation. Our results further refine the relationship between RNA methylation and pigmentation.

## MATERIALS AND METHODS

### Animals and sample collection

All related experiments involving sheep were conducted in strict compliance with the relevant guidelines set by the Ethics Committee of Tongren University, China (Approval ID: TREDU2022-076).

The sheep were provided by Taigu Haihong Animal Hus bandry Co., Ltd. (Taigu, China). Three 1-year-old Small-tailed Han Sheep of similar size with black and white fleece were selected. The white and adjacent black hairs were cut off, and the underlying skin was removed separately with a skin biopsy borer (the diameter was 1 cm). Five pieces of skin per sheep were collected for each color, placed into centrifuge tubes (1.5 mL) storing in liquid nitrogen.

### MeRIP-seq and RNA-seq library preparation

Total RNA from 6 samples (one skin piece per sheep for each color) was separated using TRIzol Reagent (Thermo Fisher Scientific, Waltham, MA, USA) and genomic DNA was eliminated by DNase I (Roche Diagnostics, Chicago, IL, USA) according to the manufacturer’s instructions. The purity and concentration of RNA were evaluated by the NanoDrop ND-1000 system (Thermo Fisher Scientific, Wilmington, DE, USA). The integrity of RNA was confirmed using agarose gel electrophoresis. The purified RNA (25 μg) was fragmented at 70°C for 6 min using RNA Fragmentation Buffer (100 mM ZnCl_2_, 100 mM TrisHCl). Most fragmented RNA (98%) was incubated with m^6^A-specific antibody (Synaptic Systems, Gottingen, Germany), and collected for immunoprecipitation (IP) and the rest (2%) for IP control (Input). The m^6^A-enriched RNA and input RNA (2 μL) were reversed to cDNA, second-strand DNA was synthesized, added with dUTP and A-base, and finally, a library was formed by polymerase chain reaction (PCR) amplification. Then the obtained libraries were sequenced on an Illumina Novaseq 6000 platform.

### Bioinformatics analysis of RNA-seq and MeRIP-seq and functional enrichment analysis

The raw data was filtered by Trimmomatic software (v0.32) (parameter set to ILLUMINACLIP:fasta.file:2:30:10 LEADING: 3 TAILING:3 SLIDINGWINDOW:4:15 MINLEN:36 CROP: 150) to obtain clean reads, which then were mapped to the reference genome of sheep (Oar_v4.0) using STAR software (v2.5.1b). Uniquely mapped reads were assembled and quantitated using StringTie (v2.1.1). The differentially expressed (DE) lncRNAs (p<0.05, |log_2_FC| ≥1) were screened using the DESeq2 algorithm.

The peaks were determined by ‘MetPeak’ of R software package (PEAK_CUTOFF_P = 0.05, FOLD_ENRICHMENT = 1) and visualized by IGV software (https://www.igv.org). The peak density plot in CDS, 5′UTR and 3′UTR was calculated by the ‘guitar’ R software package. The annotation of peaks was performed by ChIPseeker (https://bioconductor.org/packages/ChIPseeker). The motif was analyzed by HOMER (https://homer.ucsd.edu/homer/motif) software. Differentially methylated lncRNAs (DM lncRNAs) (p<0.05, |Log_2_FC| ≥1) of black skin vs white skin were analyzed using MeTDiff software. The mRNAs located 100 kb of lncRNAs upstream and downstream served as cis-target genes. The functions of the lncRNAs were predicted based on cis-target genes by the DAVID database (https://david.abcc.ncifcrf.gov/) and the Kyoto encyclopedia of genes and genomes (KEGG) database (https://www.kegg.jp/).

### Establishment of a lncRNA-mRNA network

To identify the key lncRNAs related to pigmentation in sheep skin, we selected the cis-target genes enriched in gene ontology (GO) terms and KEGG pathways that involved in pigmentation, of which corresponding DM lncRNAs and DE lncRNAs were obtained. Subsequently, the functions of cis-target genes of DM lncRNAs and DE lncRNAs were verified by NCBI PubMed (https://pubmed.ncbi.nlm.nih.gov/). The DM lncRNAs, DE lncRNAs and their cis-target genes co-expression networks were constructed with PPI and were visualized by Cytoscape 3.7.1 (https://cytoscape.org/, USA).

### MeRIP-qPCR and RT-qPCR

For each sample, 1 μg IP RNA and input RNA were prepared as mentioned above for “MeRIP-seq and RNA-seq Library Preparation”, but the total RNA didn’t require fragmented. cDNA was synthesized using PrimeScript RT Master Mix (Perfect Real Time) (TAKARA, Dalian, China). Thereafter, methylated-RNA immunoprecipitation quantitative PCR (MeRIP-qPCR) and quantitative real-time PCR (RT-qPCR) were performed using TB Green Fast qPCR Mix (TAKARA, China) on LightCycler 480II (Roche, Basel, Switzerland). The relative expression of lncRNAs was represented using 2^−ΔΔCt^. β-Actin acted as reference genes. The primer information is listed in Table 1.

### Statistical analysis

All statistical analyses for lncRNA expression levels were performed by SPSS 25 software (IBM, NY, USA). GraphPad Prism 8.0.2 software (GraphPad Software, San Diego, CA, USA) was used to generate histogram plots. The results were shown as means±standard deviation (SD). Statistical significance is defined when p values are less than 0.05. p<0.01 and p< 0.05 were marked as ** and *, respectively.

## RESULTS

### Dynamic changes in the lncRNA transcriptome in black and white sheep skin

**Figure 1:**
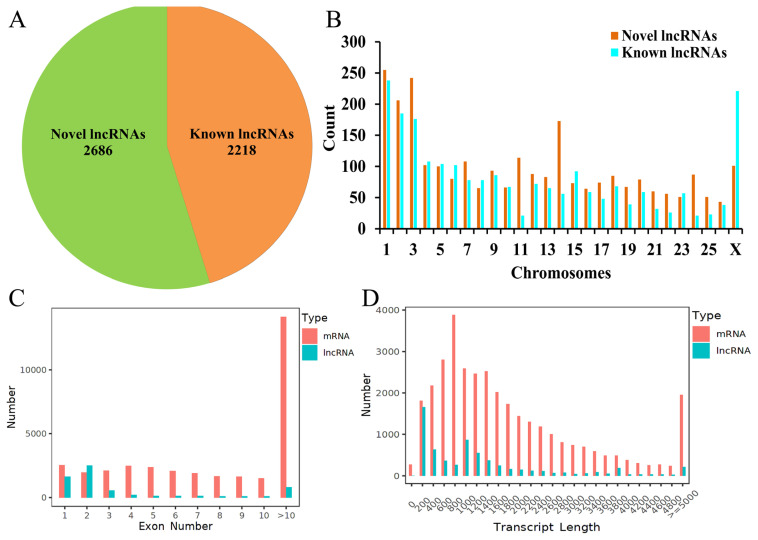
Identification and characterization of lncRNAs identified in black and white sheep skin. (A) The proportion of novel lncRNAs and known lncRNAs in sheep. (B) Chromosomal distribution of lncRNAs. (C) Distribution of exon numbers in lncRNAs and mRNAs. (D) Distribution of transcript lengths in lncRNAs and mRNAs.

To explore the function of lncRNA in pigmentation, the lncRNA profiles were extracted from the input library of black and white sheep skin. A total of 371,388,236 raw reads were obtained from six samples. Subsequently, low-quality reads and adapters were filtered out and a total of 321,835,118 clean reads were aligned to the reference genome of sheep (Oar_v4.0), accounting for 86.66% of the raw reads. A total of 4,904 lncRNAs (2,218 known lncRNAs and 2,686 novel lncRNAs) were obtained from the sheep skin specimens (Figure 1A). The lncRNAs were distributed on all 26 chromosomes, especially on chromosomes 1, 2, 3, X (Figure 1B). The transcript length and exon number of most lncRNAs were ≤2,000 bp and ≤3, respectively (Figure 1C, 1D). It is consistent with mRNAs.

**Figure 2:**
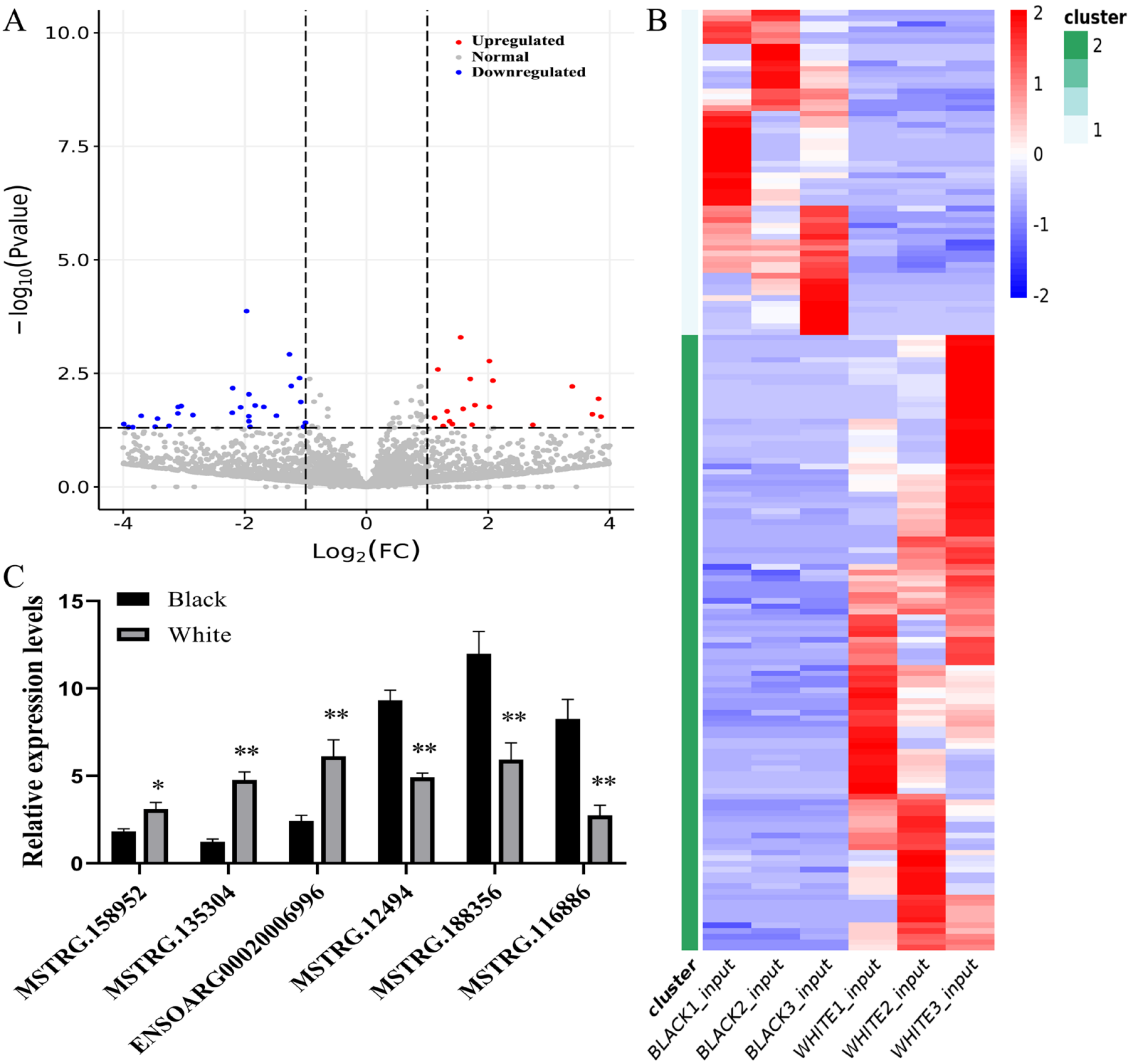
Screening analysis of DE lncRNAs in black vs white sheep skin. (A) Volcano plot of DE lncRNAs in black vs white sheep skin. The red and blue dots represent up-regulated and down-regulated lncRNAs in black sheep skin, respectively. The grey dots represent stably expressed lncRNAs in sheep skin. (B) Heatmap of DE lncRNAs in black vs. white sheep skin. Red color indicates highly expressed genes and dark blue color indicates low-expressed genes. (C) The relative expression of three down-regulated and three up-regulated DE lncRNAs. DE, differentially expressed. ** p<0.01, * p<0.05.

To screen the lncRNAs regulating pigmentation in sheep skin, DE lncRNAs were identified between black and white skin. As a result, 168 DE lncRNAs were screened out, among which 110 were down-regulated and 58 were up-regulated in black sheep skin (Figure 2A; Supplementary Table S1). Table 2 exhibits the top 10 significantly up-regulated and down-regulated lncRNAs. The heatmap shows that DE lncRNAs were significantly different between black and white skin, while their expression patterns were similar in skin samples of the same color (Figure 2B).

To verify the reliability of the results of RNA-seq, qRT-PCR was used to detect the expression levels of 6 randomly selected DE lncRNAs (3 down-regulated and 3 up-regulated lncRNAs) between black and white sheep skin. The levels of MSTRG.158952, MSTRG.135304 and ENSOARG0002000 6996 were significantly decreased in black skin vs white skin (p<0.05), while the levels of MSTRG.12494, MSTRG.188356 and MSTRG.116886 showed the opposite trend (p<0.01). These results verified the accuracy of RNA-seq (Figure 2C).

**Figure 3:**
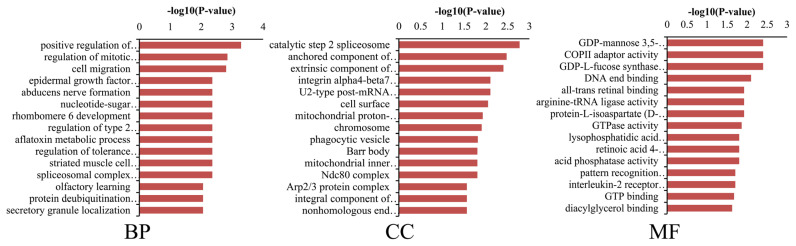
Gene ontology enrichment of differentially expressed lncRNAs in black vs white sheep skin.

**Figure 4:**
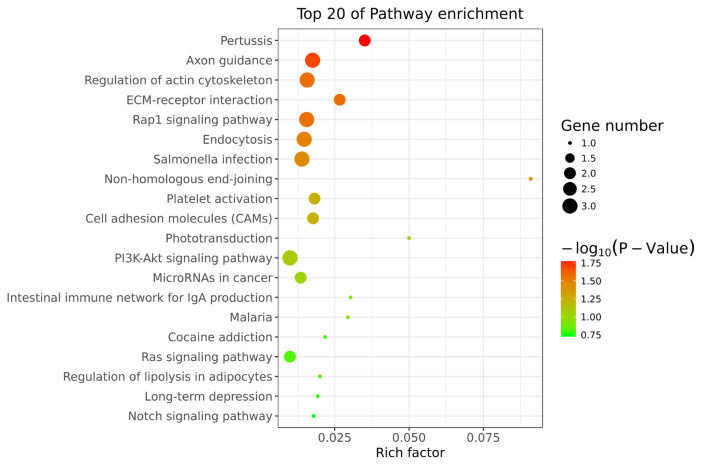
Kyoto encyclopedia of genes and genomes pathway analysis of differentially expressed lncRNAs in black vs white sheep skin.

The cis-target genes of DE lncRNAs were selected based on co-location and their functions were analyzed using the DAVID and KEGG databases. DE lncRNAs significantly enriched in GO terms, including positive regulation of exosomal secretion, epidermal growth factor catabolic process, keratinocyte differentiation, positive regulation of ERK1 and ERK2 cascade, which may involve in pigmentation (Figure 3). Meanwhile, DE lncRNAs significantly enriched in various pathways, including Pertussis, Axon guidance, Regulation of actin cytoskeleton, ECM-receptor interaction, Rap1 signaling pathway, Endocytosis, Salmonella infection, and non-homologous end-joining (p<0.05), which may play important role in pigmentation (Figure 4). In conclusion, we identified numerous novel lncRNAs and DE lncRNAs in two colors of sheep skin and focused on the potential lncRNAs associated with pigmentation.

### Overall features of lncRNAs m^6^A methylation in black and white sheep skin

**Figure 5:**
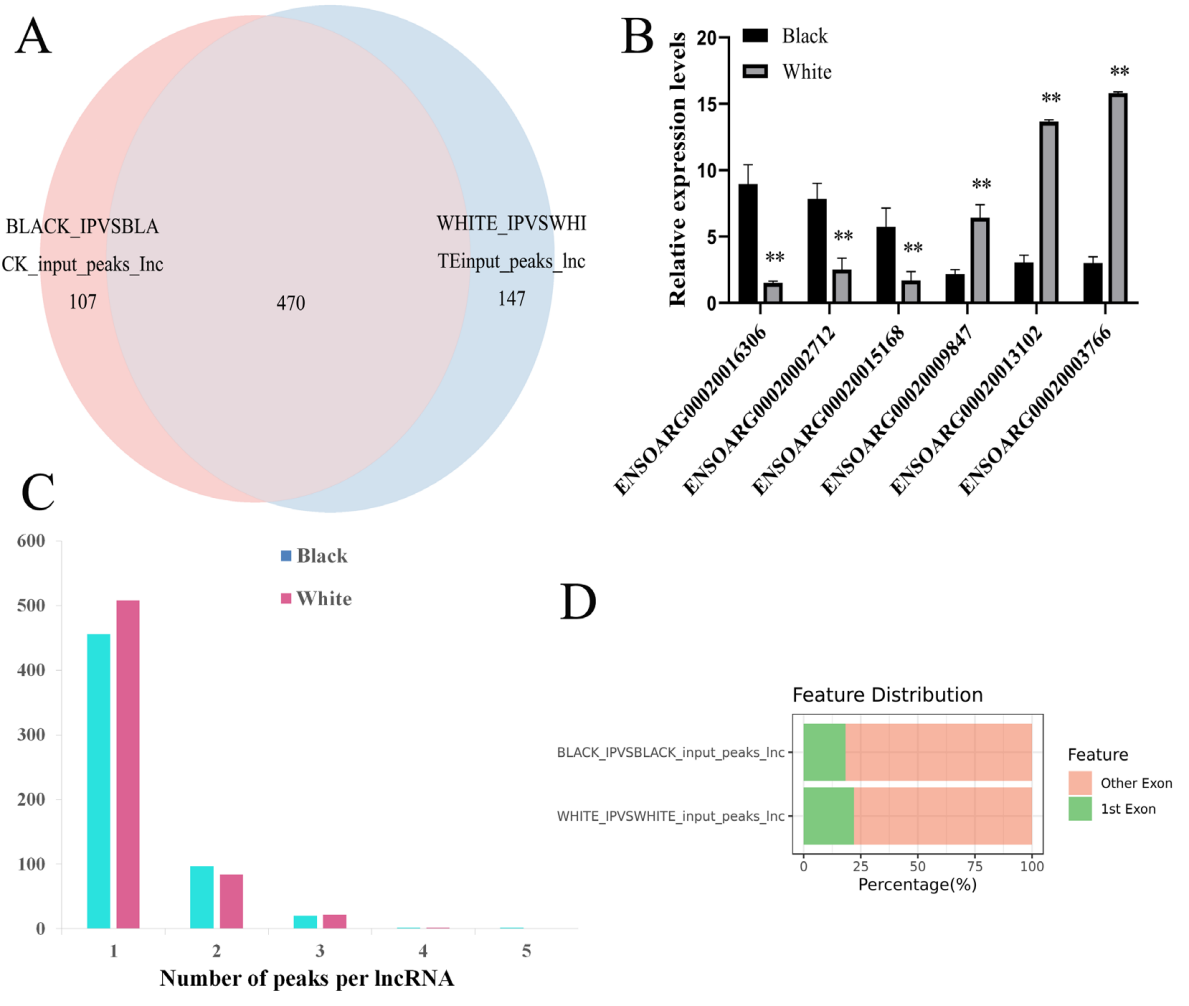
Characterization of lncRNAs m^6^A methylation in black and white sheep skin. (A) Number of m^6^A peaks found in black and white sheep skin. (B) MeRIP-qPCR validated 6 m6A-methylated lncRNAs. (C) Distribution of the peak number of m^6^A-modified lncRNAs. (D) Distribution of m^6^A peaks in the exons. MeRIP-qPCR, methylated-RNA immunoprecipitation quantitative polymerase chain reaction. ** p<0.01.

To reveal the m^6^A methylation profile of lncRNAs expressed in black and white sheep skin, MeRIP-seq was performed. The average properly paired mapped reads was 26,793,655 per sample, with the uniquely mapping rate above 71.78%. In total, we identified 577 m^6^A peaks in 243 expressed lncRNAs in black sheep skin and 617 m^6^A peaks in 254 expressed lncRNAs in white sheep skin (Supplemental Table S2). Among them, 470 peaks were m^6^A-modified in the two color skin, and 107 and 147 peaks were only m^6^A-modified in black skin and white skin, respectively (Figure 5A). There were 20 significantly different peaks in black vs white skin with p<0.05, and |log_2_FC| ≥1. Compared with white skin, 8 peaks were upregulated in black skin, which involved 8 differentially methylated lncRNAs (DM lncRNAs), while 12 peaks were downregulated in black skin, which involved 11 DM lncRNAs (Table 3).

MeRI P-qPCR analysis showed that, compared with white sheep skin, the level of m6A-modified ENSOARG00020016 306, ENSOARG00020002712, and ENSOARG00020015168 were increased and the level of m^6^A-modified ENSOARG 00020003766, ENSOARG00020013102 and ENSOARG000 20009847 were decreased in black sheep skin (p<0.01) (Figure 5B), which was similar to MeRIP-seq.

**Figure 6:**
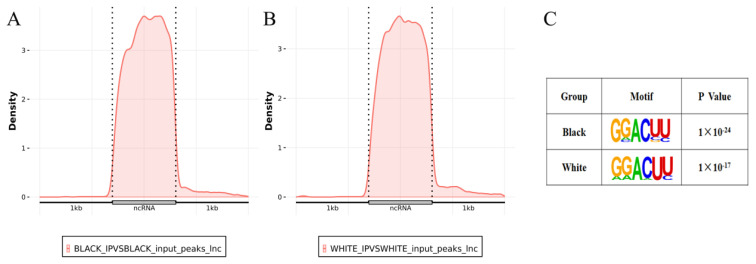
(A, B) Distribution of m^6^A peaks in the three regions of lncRNAs in black and white sheep skin. (C) The enriched consensus motif of m^6^A peaks in lncRNAs.

In sheep skin, most lncRNAs contained 1 to 2 m ^6^A peaks (Figure 5C) and most m^6^A peaks distributed in exons (Figure 5D), especially in the last exon, a small amount at the 5′end and a slight increase at the 3′ end (Figure 6A, 6B).

To identify the common sequence elements on the m ^6^A peaks of lncRNAs, HOMER software was used to determine a consensus motif. We found that “GGACU” was the enriched motif in both black and white sheep skin (Figure 6C), in accord with the well validated consensus motif “RRACH” (R = A or G, H = A, C or U).

### Function analysis of DM lncRNAs

**Figure 7:**
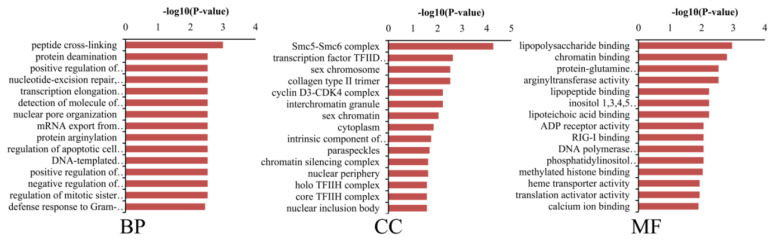
Gene ontology enrichment of differentially methylated (DM) lncRNAs in black vs white sheep skin.

**Figure 8:**
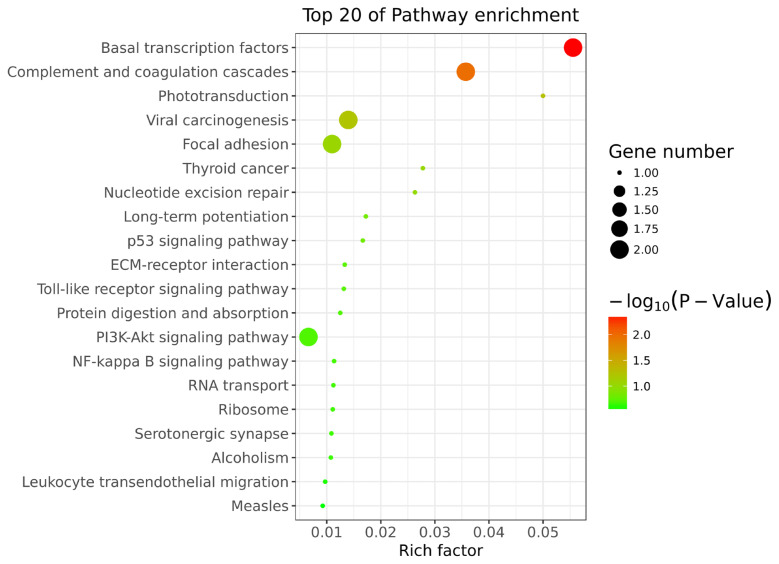
Kyoto encyclopedia of genes and genomes pathway of differentially methylated (DM) lncRNAs in black vs white sheep skin.

To explore the functions of 19 DM lncRNAs, the cis-target genes were predicted and used to perform GO and KEGG pathway enrichment. A total of 48 cis-target genes were enriched in 382 GO terms, among which 116 biological process, 21 cellular component, and 31 molecular function GO terms were significantly enriched (p<0.05). These included peptide cross-linking, protein deamination, Smc5-Smc6 complex, transcription factor TFIID complex, lipopolysaccharide binding, chromatin binding, etc. (Figure 7). KEGG pathway analysis revealed 37 enriched pathways; among them, basal transcription factors and complement and coagulation cascades were significantly enriched (p<0.05) (Figure 8). Furthermore, Phototransduction, ECM-receptor interaction, toll-like receptor signaling pathway, PI3K-Akt signaling pathway, NF-kappa B signaling pathway, Wnt signaling pathway, Rap1 signaling pathway, and cAMP signaling pathway may participate in skin pigmentation.

### Conjoint analysis of MeRIP-seq and RNA-seq data

**Figure 9:**
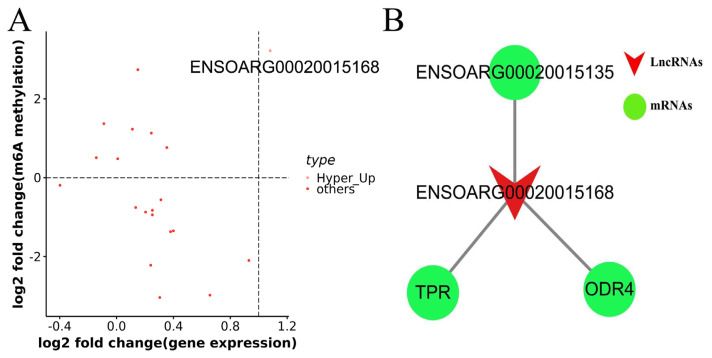
Conjoint analyses of MeRIP-Seq and RNA-Seq data. (A) Distribution of lncRNAs in both expression levels and m6A methylation levels in black vs white sheep skin. Pink dot represents Hyper_Up lncRNAs. Red dots represent other lncRNAs. (B) Network of the hyper lncRNA and its cis-target genes.

To further excavate the role of m^6^A lncRNAs, MeRIP-seq and RNA-seq data were analyzed conjointly. Our results showed that ENSOARG00020015168 was the unique lncRNA that was highly expressed and highly methylated (Hyper-Up) in black sheep skin (Figure 9A). This implies that ENSOARG00020015168 might participate in the regulation of pigmentation in sheep skin. There were three cis-target genes of ENSOARG00020015168, namely, *TPR*, *ODR4*, and ENSOARG00020015135 (Figure 9B). The GO enrichment and KEGG pathway of TPR was mainly involved in RNA transport, nuclear, response to heat stress, mitosis, and thyroid cancer, RNA transport, pathways in cancer, amyotrophic lateral sclerosis, respectively. However, the functions of ODR4 and ENSOARG00020015135 were not enriched.

### Identification of key pigmentation-related lncRNAs

**Figure 10:**
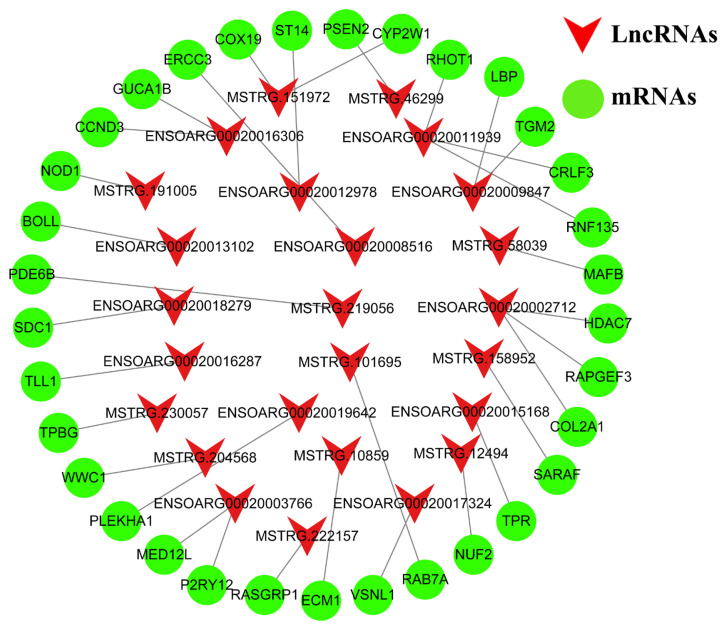
LncRNA-mRNA network involved in pigmentation visualized using Cytoscape.

To identify the key lncRNAs related to pigmentation in sheep skin, the GO terms and KEGG pathways of DM lncRNAs and DE lncRNAs involving in pigmentation were selected, and the corresponding cis-target genes and lncRNAs were listed. Subsequently, the functions of cis-target genes of DM lncRNAs and DE lncRNAs were verified using NCBI PubMed. After removing duplicates, we picked out 25 lncRNAs and 33 cis-target genes and constructed the lncRNA-mRNA network (Figure 10). Among them, MSTRG.46299-PSEN2, ENSOARG00020016306-CCND3, ENSOARG00020002712-COL2A1, and ENSOARG00020008516-ERCC3 might be key candidates in the regulation of pigmentation.

The expression level of key cis-target mRNAs (PSEN2, CCND3, COL2A, ERCC3, and TPR) in black and white sheep skin was obtained from RNA-seq of previous studies and listed in Table 4. The results showed that the abundances of PSEN2, CCND3, COL2A and ERCC3 in black sheep skin were higher than those in white sheep skin, especially CCND3 and COL2A (p<0.05), and the expression trend of TPR was opposite.

## DISCUSSION

Although some lncRNAs have been proven to participate in the regulation of pigmentation process, such as H19, UCA1, SPRIGHTLY, TUG1, TCONS_00049140, ENST00000606533, SRA, etc. [14–20], their number is small, and there are still many lncRNAs related to pigmentation waiting to be discovered. In this study, we detected the lncRNA profiles in black and white sheep skin and obtained 4,904 lncRNAs, among which 2,686 novel lncRNAs were identified. This result broadens the annotation of the sheep lncRNAs. At the same time, the transcript length, exon number and expression of novel lncRNAs consisted with previously known lncRNAs but differed from the mRNAs, which was similar to pigs and goats [13,32]. We identified 168 DE lncRNAs (110 down-regulated and 58 up-regulated) between black and white sheep skin, which might be key candidate lncRNAs related to pigmentation.

Studies have demonstrated that m ^6^A is the most universal post-transcriptional modification of mRNA in various species and plays a key role in growth, reproduction, pigmentation, fat metabolism, nerve development, tumor invasion, immune responses, and other physiological processes by regulating the expression of mRNAs [30,31]. The role of m^6^A methylation of lncRNAs has received much attention from researchers. The m^6^A methylation of lncRNAs in skeletal muscles of pig and Cattle-Yak has been excavated to find new candidate lncRNAs of muscle-fiber-type conversion and muscle development [32,33]. The change of m^6^A methylation of lncRNAs in IPEC-J2 cells induced by *Clostridium perfringens* beta2 toxin has been described [34]. However, the role of m^6^A methylation of lncRNAs in pigmentation remains unclear. In this study, we identified 577 and 617 m^6^A peaks in 243 and 254 expressed lncRNAs in black and white sheep skin, respectively. The average peak number was 2.37 to 2.43, indicating that m6A modification widely existed in lncRNAs. In addition, in accord with the typical m6A consensus motif “RRACH” (R = A or G, H = A, C or U), “GGACU” motif enriched in both black and white sheep skin, which was similar with previous studies [27,28,35,36]. The adenosine in the GGACU consensus motif can be methylated by the METTL3-METTL14 complex to form m6A, which can also be removed from these concordance sites by enzymes, thereby mediating gene expression [37]. In the current study, the differences of sheep skin color may be caused by the m^6^A methylation of “GGACU” motif of lncRNAs. The m^6^A peaks were mainly distributed CDS of the lncRNAs, a small amount at the 5′end and a slight increase at the 3′ end, which agreed with previous studies [32]. It means that the m^6^A peaks of lncRNA identified in this study are credible. M^6^A modification of mRNA affects its stability, splicing, translation, and nuclear transport [30]. M^6^A modification of lncRNAs also can affect their expression, such as METTL3 mediated the expression of LINC00958 and lncRNA EN_42575 [38,39]. In the present study, a total of 20 overlapping lncRNAs were found in DE lncRNAs and DM lncRNAs. Among them, 7 lncRNAs had the same expression trend as the methylation trend, while 13 lncRNAs had the opposite trend, indicating that m^6^A levels of most lncRNAs were negatively correlated with their expression levels. It means that m^6^A methylation may negatively regulate the expression of these lncRNAs. M^6^A reader proteins YTHDF2 can mediate RNA decay, while YTHDF1/3, YTHDC1, and IGF2BP1/2/3 can enhance the stability of RNA [40,41]. Therefore, we speculate that the lncRNAs whose expression levels are negatively regulated by m^6^A may be mainly identified and bound by YTHDF2, thereby decaying their expression. However, further research is needed to verify this hypothesis.

A large number of studies have suggested that the biologi cal function of lncRNA could be predicted using cis-genes [42]. Therefore, the function of DE lncRNAs and DM lncRNAs were predicted based on their cis-target genes using the DAVID and KEGG databases. Finally, we identified 73 lncRNAs and 104 cis-target genes that might be involved in the pigmentation of sheep skin. Among them, MSTRG.46299-PSEN2, ENSOARG00020016306-CCND3, ENSOARG0002 0002712-COL2A1, and ENSOARG00020008516-ERCC3 might be key candidates in the regulation of pigmentation because PSEN2, CCND3, COL2A1, and ERCC3 participate in melanocyte proliferation or epidermal pigmentation or melanin synthesis. For example, in zebrafish embryos, reduced Psen2 activity leads to a decrease of melanocyte number in the trunk [43]. Meanwhile, the mutation of *Psen2* gene also makes skin pigmentation gradually disappear with growth [44]. Cyclin D3 (CCND3) is involved in the progression and proliferation of the G1-S cell cycle of melanoma cells and expressed in melanocytes. Therefore, CCND3 may also be involved in melanocyte proliferation [45]. Type II collagen (COL2A1) is a key secretory regulator of melanogenesis and epidermal pigmentation during UV-induced epidermal pigmentation [46]. As shown by *in vitro* experiments, siRNA interferes with the expression of ERCC3, resulting in decreased tyrosinase production capacity of human melanocytes [47].

In conclusion, we identified lncRNAs and m ^6^A-methylation of lncRNAs in sheep skin, screened out DE lncRNAs and DM lncRNAs between black and white sheep skin which may be key candidate lncRNAs in pigmentation. Our study supplements the lncRNAs resources of sheep, opening a new avenue to study the function and mechanism of lncRNAs modified by m^6^A as well as RNA epigenetics in pigmentation.

## Figures and Tables

**Table 1:** Primer sequences of lncRNAs

lncRNAs	Primer sequence (5′-3′)	Product length (bp)	Type
MSTRG.158952	F: ACCAGCTTCATCCCGAAGTC	247	RT-qPCR
	R: GTGGGTCTCACCAGAACTCC		
MSTRG.135304	F: GGATCAGCAGCAACAAAGCAA	83	RT-qPCR
	R: GTCGCACACCTACCCTAACA		
ENSOARG00020006996	F: CCTCTCAGTGTGAAGGGCAG	101	RT-qPCR
	R: ACGACAGACGACATTGGCAT		
MSTRG.12494	F: ACGTGTTTGGCTTTGGAGATGT	100	RT-qPCR
	R: GACAGCCCCAAGTCCTCCTTT		
MSTRG.188356	F: TTCCTTGTGGCTTAGCTGGT	110	RT-qPCR
	R: CAAGAGGCAGGGTAGCCTTT		
MSTRG.116886	F: TGGCAGTCAGACCTGGTAGA	78	RT-qPCR
	R: AGTCTGCCAAAACCTGAACCA		
ENSOARG00020016306	F: AGGAGACTGAGAAGGTCCCC	248	MeRIP-qPCR
	R: CTTGTCTTCCTCCGGCTTGT		
ENSOARG00020002712	F: GATCTCCTGACTCTGGCAGC	254	MeRIP-qPCR
	R: CATGACTGAGGCCTTGAGGG		
ENSOARG00020015168	F: TTGTAACAGAGGCGGCAACT	285	MeRIP-qPCR
	R: TCGAAGTGGGCTTTTTGGGT		
ENSOARG00020003766	F: AGCTTATCCCCCATCACCCT	168	MeRIP-qPCR
	R: CACTTGGGCACTTTCAGCAC		
ENSOARG00020013102	F: TCACACCCCACTCGAGTACA	205	MeRIP-qPCR
	R: TCTGTTGTGACAGGGAGGGA		
ENSOARG00020009847	F: CGTCTGCCTCACGATCAAGT	217	MeRIP-qPCR
	R: ATCCACAAACTCTGGGCTGG		
β-actin	F: GCAGGAGTACGATGAGTCCG	238	MeRIP-qPCR / RT-qPCR
	R: AACCGACTGCTGTCCCCTT		

RT-qPCR, quantitative real-time polymerase chain reaction; MeRIP-qPCR, methylated-RNA immunoprecipitation quantitative polymerase chain reaction.

**Table 2:** Top 10 significantly up-regulated and down-regulated lncRNAs (black vs white)

Chromosome	ChromStart	ChromEnd	lncRNA_name	p-value	Strand	Log_2_FC	Regulation
26	28655655	28662081	MSTRG.158952	2.81E-07	−	−8.3828	down
21	37488901	37490858	MSTRG.135304	1.35E-05	+	−8.05744	down
3	129434127	129491471	ENSOARG00020006996	1.39E-05	+	−7.97717	down
1	76042121	76060476	MSTRG.7779	5.54E-05	−	−7.87104	down
18	21948171	21964423	ENSOARG00020021751	2.70E-05	−	−7.67419	down
9	15338970	15339776	MSTRG.239209	5.67E-05	+	−7.60017	down
17	5781916	5782557	MSTRG.82249	3.84E-05	−	−7.57951	down
17	60092485	60103158	MSTRG.86588	0.010517	−	−7.45528	down
2	264636036	264636777	MSTRG.126245	0.000204	+	−7.3965	down
2	114961670	115014756	MSTRG.112600	0.012261	−	−7.36238	down
8	31245830	31262128	MSTRG.231992	0.013362	−	7.387439	up
3	228593008	228593520	MSTRG.182902	0.000296	−	7.422393	up
1	122302257	122305820	MSTRG.12494	0.009963	−	7.453012	up
15	35639980	35688245	MSTRG.70109	0.000616	−	7.486254	up
5	34276673	34276900	MSTRG.199719	0.00702	+	7.681704	up
4	46223839	46226430	MSTRG.188356	0.00627	+	7.795649	up
1	220731438	220755947	MSTRG.21517	0.005871	−	7.905694	up
3	3366120	3369275	MSTRG.161430	0.003987	+	8.01902	up
2	161881064	161883035	MSTRG.116886	0.001777	+	8.210925	up
2	142519666	142553890	MSTRG.114993	0.001645	−	8.51941	up

**Table 3:** Top 10 significantly up-regulated and down-regulated m6A peaks of lncRNAs (black vs white)

Chromosome	ChromStart	ChromEnd	Peak_name	p-value	Strand	Log_2_FC	Regulation
1	257875778	257902264	ENSOARG00020003766	3.8×10^−5^	+	−17.5	down
2	213805024	214255402	ENSOARG00020013102	3.2×10^−7^	+	−16.5	down
3	40599038	40599886	ENSOARG00020006215	9.5×10^−8^	+	−15.7	down
13	69905937	69906438	ENSOARG00020009847	9.1×10^−5^	−	−11.4	down
8	37133158	37133756	ENSOARG00020004408	1.2×10^−5^	−	−11.2	down
20	21742623	21743323	ENSOARG00020013553	3.5×10^−7^	−	−9.71	down
11	45561607	45561856	ENSOARG00020011939	2.1×10^−4^	+	−4.33	down
20	21735780	21737193	ENSOARG00020013553	5.5×10^−6^	−	−3.63	down
4	12166268	12166419	ENSOARG00020015965	4.1×10^−8^	−	−2.8	down
8	10962183	10962284	ENSOARG00020015784	7.6×10^−5^	−	−2.5	down
3	27170853	27171053	ENSOARG00020017324	5.0×10^−16^	+	−2.4	down
12	79562199	79562300	ENSOARG00020022464	1.0×10^−3^	−	−2.25	down
10	38385162	38385462	ENSOARG00020005446	7.1×10^−5^	−	1.57	up
22	45256625	45256975	ENSOARG00020019642	1.9×10^−3^	+	2.23	up
2	128017581	128017830	ENSOARG00020008516	6.0×10^−3^	+	2.33	up
8	90274609	90274809	ENSOARG00020005956	4.2×10^−4^	+	2.63	up
17	2331763	2332085	ENSOARG00020016287	1.7×10^−3^	+	2.86	up
12	72406857	72406958	ENSOARG00020015168	8.1×10^−5^	+	3.33	up
3	148874470	148874716	ENSOARG00020002712	5.2×10^−6^	+	3.56	up
20	17587109	17587858	ENSOARG00020016306	5.6×10^−3^	−	6.72	up

**Table 4:** The expression of cis-target mRNAs of lncRNAs (black vs white)

Chromosome	ChromStart	ChromEnd	Gene_name	p-value	Strand	Log_2_FC	Regulation
12	32537941	32558470	*PSEN2*	9.7×10^−3^	+	3.9	up
20	17452108	17546717	*CCND3*	0.04	−	1.33	up
3	148719985	148749913	*COL2A1*	0.03	+	1.93	up
2	127897184	127950203	*ERCC3*	0.03	+	1.1	up
12	72413463	72476857	*TPR*	0.02	−	−1.22	down

## Data Availability

All datasets used in this study are available from the corresponding author on reasonable request.

## SUPPLEMENTARY MATERIAL

**Supplementary Table S1**. Differently expressed lncRNAs in black and white skin of sheep

## References

[1] Bhat B, Singh A, Iqbal Z (2019). Comparative transcriptome analysis reveals the genetic basis of coat color variation in Pashmina goat. Sci Rep.

[2] Kuzumaki T, Matsuda A, Wakamatsu K, Ito S, Ishikawa K (1993). Eumelanin biosynthesis is regulated by coordinate expression of tyrosinase and tyrosinase-related protein-1 genes. Exp Cell Res.

[3] Gelmi MC, Houtzagers LE, Strub T, Krossa I, Jager MJ (2022). Mitf in normal melanocytes, cutaneous and uveal melanoma: a delicate balance. Int J Mol Sci.

[4] Archambault M, Yaar M, Gilchrest BA (1995). Keratinocytes and fibroblasts in a human skin equivalent model enhance melanocyte survival and melanin synthesis after ultraviolet irradiation. J Invest Dermatol.

[5] Wang Y, Viennet C, Robin S, Berthon JY, He L, Humbert P (2017). Precise role of dermal fibroblasts on melanocyte pigmentation. J Dermatol Sci.

[6] Fu C, Chen J, Lu J (2020). Roles of inflammation factors in melanogenesis. Mol Med Rep.

[7] Zhou S, Zeng H, Huang J (2021). Epigenetic regulation of melanogenesis. Ageing Res Rev.

[8] Kopp F, Mendell JTJC (2018). Functional classification and experimental dissection of long noncoding RNAs. Cell.

[9] Ji K, Fan R, Zhang J, Yang S, Dong C (2018). Long non-coding RNA expression profile in Cdk5-knockdown mouse skin. Gene.

[10] Ji K, Zhang J, Fan R, Yang S, Dong C (2018). ifferential expression of lncRNAs and predicted target genes in normal mouse melanocytes and B16 cells. Exp Dermatol.

[11] Ji KY, Zhao YW, Wen RJ, Ibrar MK, Zhang YH (2022). A genome-wide integrated analysis of lncRNA-mRNA in melanocytes from white and brown skin hair boer goats (Capra aegagrus hircus). Front Vet Sci.

[12] Zhao B, Luo H, He J (2021). Comprehensive transcriptome and methylome analysis delineates the biological basis of hair follicle development and wool-related traits in Merino sheep. BMC Biol.

[13] Jin L, Zhao L, Hu S (2019). Transcriptional differences of coding and non-coding genes related to the absence of melanocyte in skins of bama pig. Genes (Basel).

[14] Pei S, Huang J, Chen J (2018). UVB-inhibited H19 activates melanogenesis by paracrine effects. Exp Dermatol.

[15] Kim NH, Choi SH, Kim CH, Lee CH, Lee TR, Lee AY (2014). Reduced MiR-675 in exosome in H19 RNA-related melanogenesis via MITF as a direct target. J Invest Dermatol.

[16] Zhao W, Mazar J, Lee B (2016). The long noncoding RNA SPRIGHTLY regulates cell proliferation in primary human melanocytes. J Invest Dermatol.

[17] Jiang L, Huang J, Hu Y (2021). Identification of the ceRNA networks in α-MSH-induced melanogenesis of melanocytes. Aging (Albany NY).

[18] Pei S, Chen J, Lu J (2020). The long noncoding RNA UCA1 negatively regulates melanogenesis in melanocytes. J Invest Dermatol.

[19] Fu C, Chen J, Lu J (2019). Downregulation of TUG1 promotes melanogenesis and UVB-induced melanogenesis. Exp Dermatol.

[20] Ho JC, Lee CH, Hong CH (2020). Targeting steroid receptor RNA activator (SRA), a long non-coding RNA, enhances melanogenesis through activation of TRP1 and inhibition of p38 phosphorylation. PLoS One.

[21] Zeng Q, Wang Q, Chen X (2016). Analysis of lncRNAs expression in UVB-induced stress responses of melanocytes. J Dermatol Sci.

[22] Deng X, Chen K, Luo GZ (2015). Widespread occurrence of N 6-methyladenosine in bacterial mRNA. Nucleic Acids Res.

[23] Lence T, Soller M, Roignant JY (2017). A fly view on the roles and mechanisms of the m6A mRNA modification and its players. RNA Biol.

[24] Zhao BS, Roundtree IA, He C (2017). Post-transcriptional gene regulation by mRNA modifications. Nat Rev Mol Cell Biol.

[25] Yue H, Nie X, Yan Z, Weining S (2019). N6-methyladenosine regulatory machinery in plants: composition, function and evolution. Plant Biotechnol J.

[26] Oerum S, Meynier V, Catala M, Tisné C (2021). A comprehensive review of m6A/m6A mRNA methyltransferase structures. Nucleic Acids Res.

[27] Zhang J, Yang Q, Yang J (2021). Comprehensive analysis of transcriptome-wide m(6)A methylome upon clostridium perfringens beta2 toxin exposure in porcine intestinal epithelial cells by m(6)A sequencing. Front Genet.

[28] Lu Z, Liu J, Yuan C (2021). m(6)A mRNA methylation analysis provides novel insights into heat stress responses in the liver tissue of sheep. Genomics.

[29] Dahal U, Le K, Gupta M (2019). RNA m6A methyltransferase METTL3 regulates invasiveness of melanoma cells by matrix metallopeptidase 2. Melanoma Res.

[30] Wang ZY, Li P, Hu JP, Xu Q, Zhang CY (2023). Construction of a single-molecule biosensor for antibody-free detection of locus-specific N 6-methyladenosine in cancer cells and tissues. Anal Chem.

[31] Zhao Y, Meng J, Song X, An Q (2023). m6A mRNA methylation analysis provides novel insights into pigmentation in sheep skin. Epigenetics.

[32] Wang S, Tan B, Xiao L (2022). Comprehensive analysis of long noncoding RNA modified by m6A methylation in oxidative and glycolytic skeletal muscles. Int J Mol Sci.

[33] Huang C, Dai R, Meng G (2022). Transcriptome-wide study of mRNAs and lncRNAs modified by m6A RNA methylation in the longissimus dorsi muscle development of cattle-yak. Cells.

[34] Yang J, Yang Q, Zhang J (2021). N6-methyladenosine methylation analysis of long noncoding RNAs and mRNAs in IPEC-J2 cells treated with clostridium perfringens beta2 toxin. Front Immunol.

[35] He S, Wang H, Liu R (2017). mRNA N6-methyladenosine methylation of postnatal liver development in pig. PLoS One.

[36] Qin Z, Wang W, Ali MA (2021). Transcriptome-wide m(6)A profiling reveals mRNA post-transcriptional modification of boar sperm during cryopreservation. BMC Genomics.

[37] Melzer ME, Sweedler JV, Clark KD (2022). Rapid determination of RNA modifications in consensus motifs by nuclease protection with ion-tagged oligonucleotide probes and matrix-assisted laser desorption ionization mass spectrometry. Genes (Basel).

[38] Zuo X, Chen Z, Gao W (2020). M6A-mediated upregulation of LINC00958 increases lipogenesis and acts as a nanotherapeutic target in hepatocellular carcinoma. J Hematol Oncol.

[39] Yang J, Yang Q, Huang X (2023). METTL3-mediated LncRNA EN_42575 m6A modification alleviates CPB2 toxin-induced damage in IPEC-J2 cells. Int J Mol Sci.

[40] Zhu T, Roundtree IA, Wang P (2014). Crystal structure of the YTH domain of YTHDF2 reveals mechanism for recognition of N6-methyladenosine. Cell Res.

[41] Zhu S, Wang JZ, Chen D (2020). An oncopeptide regulates m(6)A recognition by the m(6)A reader IGF2BP1 and tumorigenesis. Nat Commun.

[42] Hong L, Hu Q, Zang X (2020). Analysis and screening of reproductive long non-coding RNAs through genome-wide analyses of goat endometrium during the pre-attachment phase. Front Genet.

[43] Nornes S, Newman M, Wells S, Verdile G, Martins RN, Lardelli M (2009). Independent and cooperative action of Psen2 with Psen1 in zebrafish embryos. Exp Cell Res.

[44] Jiang H, Newman M, Lardelli M (2018). The zebrafish orthologue of familial Alzheimer’s disease gene PRESENILIN 2 is required for normal adult melanotic skin pigmentation. PLoS One.

[45] Spofford LS, Abel EV, Boisvert-Adamo K, Aplin AE (2006). Cyclin D3 expression in melanoma cells is regulated by adhesion-dependent phosphatidylinositol 3-kinase signaling and contributes to G1-S progression. J Biol Chem.

[46] Li MY, Flora P, Pu H (2021). UV-induced reduction in Polycomb repression promotes epidermal pigmentation. Dev Cell.

[47] Yu M, Bell RH, Ho MM (2012). Deficiency in nucleotide excision repair family gene activity, especially ERCC3, is associated with non-pigmented hair fiber growth. PLoS One.

