# Supplementary material for: Comprehensive analysis of lncRNAs modified by m6A methylation in sheep skin
Source: Anim Biosci. 2024 May 7;37(11):1887–900. doi: 10.5713/ab.24.0039 (PMC11541038; doi:10.5713/ab.24.0039)
Supplement: Supplementary file 1 [file ab-24-0039-Supplementary-Table-1.pdf]

Table S1 Differently expressed lncRNAs in black and white skin of sheep

lncRNA_id	log2FoldChange	pvalue	Style
MSTRG.158952	-8.382799206	2.81E-07	down
MSTRG.135304	-8.057436775	1.35E-05	down
ENSOARG00020006996	-7.977168837	1.39E-05	down
MSTRG.7779	-7.871040455	5.54E-05	down
ENSOARG00020021751	-7.67419003	2.70E-05	down
MSTRG.239209	-7.600174397	5.67E-05	down
MSTRG.82249	-7.579507936	3.84E-05	down
MSTRG.86588	-7.455282354	0.010516896	down
MSTRG.126245	-7.39650079	0.000204244	down
MSTRG.112600	-7.362380436	0.012261124	down
MSTRG.116512	-7.334881771	0.000813616	down
MSTRG.229171	-7.307036897	0.015812835	down
MSTRG.184052	-7.302711741	0.017372133	down
MSTRG.191005	-7.302545012	0.013573724	down
MSTRG.31454	-7.274940443	0.014762045	down
MSTRG.101695	-7.255711218	0.014485432	down
MSTRG.151972	-7.211008646	0.001137156	down
MSTRG.250472	-7.208706286	0.016809157	down
MSTRG.208658	-7.199137189	0.016475954	down
MSTRG.227321	-7.194198168	0.017317006	down
MSTRG.58039	-7.175005237	0.018113703	down
MSTRG.141887	-7.174530677	0.018381508	down
MSTRG.94946	-7.172908554	0.000797678	down
MSTRG.47807	-7.166167507	0.01731429	down
MSTRG.235736	-7.159143294	0.017450278	down
MSTRG.64542	-7.153195873	0.001158165	down
MSTRG.38884	-7.152195657	0.017274155	down
MSTRG.234484	-7.129579856	0.000694779	down
MSTRG.131445	-7.126514031	0.018933074	down
MSTRG.229159	-7.112770526	0.018838294	down
ENSOARG00020022313	-7.092576402	0.000753234	down
MSTRG.51133	-7.081161721	0.022183804	down
MSTRG.155516	-7.067064871	0.028687273	down
ENSOARG00020009932	-7.066765344	0.02031268	down
MSTRG.230237	-6.994076447	0.03355716	down
MSTRG.182375	-6.985777842	0.000833229	down
ENSOARG00020005880	-6.94972804	0.02575409	down
MSTRG.232837	-6.947406473	0.026180874	down
MSTRG.258570	-6.939841257	0.025856252	down
MSTRG.37501	-6.926508537	0.026326813	down
MSTRG.241871	-6.920294789	0.028959972	down
MSTRG.204568	-6.910893988	0.040291486	down
MSTRG.155015	-6.875358697	0.030223466	down
MSTRG.11722	-6.872554048	0.028828838	down
MSTRG.75191	-6.86965277	0.038153016	down
ENSOARG00020012978	-6.868765727	0.039961194	down
MSTRG.96062	-6.850685483	0.030105561	down
MSTRG.145406	-6.805229787	0.034452419	down
MSTRG.21305	-6.780672771	0.036972484	down
MSTRG.152146	-6.744018562	0.039602753	down
MSTRG.56577	-6.735283981	0.003277434	down
MSTRG.76485	-6.733475169	0.044594157	down

ENSOARG00020013382	-6.731237626	0.007911843	down
MSTRG.64243	-6.716044333	0.002690021	down
ENSOARG00020025203	-6.715229779	0.040998027	down
MSTRG.196234	-6.714574471	0.038059965	down
MSTRG.151536	-6.711804355	0.003096677	down
MSTRG.39188	-6.708170044	0.049438754	down
MSTRG.53929	-6.700209671	0.046655432	down
ENSOARG00020022361	-6.652158728	0.00019487	down
MSTRG.19779	-6.614021408	0.044976945	down
ENSOARG00020012274	-6.579812468	0.046069481	down
MSTRG.75789	-6.557453919	0.049440439	down
MSTRG.155110	-6.491536661	0.005548319	down
MSTRG.60423	-6.441238887	0.009290088	down
MSTRG.162958	-6.295650811	0.01751059	down
MSTRG.245237	-6.255478142	0.015672894	down
MSTRG.174308	-6.209759016	0.02053885	down
MSTRG.202956	-6.14503345	0.024838802	down
ENSOARG00020014472	-6.006707637	0.030730207	down
ENSOARG00020025806	-5.985210607	0.043839284	down
ENSOARG00020005371	-5.79198583	0.039022847	down
ENSOARG00020000550	-5.787380046	0.034576217	down
MSTRG.164549	-5.429828184	0.016290493	down
MSTRG.46299	-5.419976268	0.023748693	down
ENSOARG00020020687	-5.153927824	0.029733347	down
MSTRG.101804	-5.023020216	0.005720386	down
MSTRG.196391	-4.870683799	0.021909974	down
MSTRG.92558	-4.861464097	0.049480256	down
MSTRG.470	-4.735255191	0.021187493	down
MSTRG.137051	-4.506829937	0.011493292	down
MSTRG.222157	-4.154849959	0.027203796	down
MSTRG.151070	-3.988938127	0.041261774	down
MSTRG.221126	-3.910957467	0.048137868	down
ENSOARG00020002803	-3.842816063	0.04841469	down
MSTRG.88669	-3.703176789	0.027253108	down
MSTRG.51173	-3.474879494	0.04737511	down
MSTRG.131013	-3.436140972	0.031312838	down
MSTRG.192257	-3.246526145	0.045534434	down
MSTRG.18992	-3.100587549	0.024081028	down
MSTRG.94947	-3.096338483	0.017385073	down
MSTRG.31560	-3.045776186	0.016605944	down
MSTRG.183855	-2.853747686	0.026197206	down
ENSOARG00020018279	-2.206573412	0.023321307	down
ENSOARG00020022125	-2.19929584	0.006707356	down
MSTRG.56405	-2.069525832	0.017705645	down
MSTRG.101497	-1.971248617	0.000134695	down
MSTRG.101779	-1.935889585	0.027796756	down
MSTRG.7786	-1.933592681	0.035805829	down
MSTRG.74961	-1.933442244	0.009145314	down
MSTRG.28423	-1.91525425	0.047490431	down
MSTRG.239060	-1.830508354	0.016171668	down
MSTRG.146346	-1.688617655	0.017365963	down
MSTRG.197028	-1.48022116	0.027128029	down
MSTRG.193685	-1.266532449	0.001203458	down
MSTRG.148174	-1.236844877	0.0059946	down

MSTRG.241046	-1.099386863	0.004018077	down
MSTRG.137000	-1.079512211	0.013498893	down
MSTRG.228991	-1.034827072	0.047157748	down
MSTRG.58391	-1.003082866	0.038462985	down
MSTRG.104304	1.120992712	0.030220188	up
ENSOARG00020012459	1.174951596	0.002595855	up
MSTRG.88151	1.260948917	0.045783654	up
MSTRG.147942	1.326790341	0.021714249	up
MSTRG.27753	1.367477761	0.035822351	up
MSTRG.90763	1.412796285	0.041395375	up
ENSOARG00020001206	1.547592145	0.000509132	up
MSTRG.147903	1.588557674	0.019152587	up
MSTRG.127856	1.706708094	0.004202355	up
ENSOARG00020005731	1.736566612	0.042783781	up
ENSOARG00020014451	1.782513675	0.015851579	up
MSTRG.247479	2.019428615	0.017394622	up
ENSOARG00020017120	2.019914845	0.001696705	up
MSTRG.26616	2.078399434	0.004552458	up
MSTRG.141391	2.735254143	0.043062824	up
MSTRG.93123	3.38253017	0.006133115	up
MSTRG.15778	3.713738439	0.025190158	up
MSTRG.53076	3.815149937	0.011478305	up
MSTRG.210010	3.855377605	0.028351195	up
MSTRG.196076	4.210809006	0.037791128	up
ENSOARG00020025805	4.84734551	0.044286473	up
MSTRG.247924	5.026989104	0.04934984	up
ENSOARG00020004429	5.151852067	0.0403502	up
ENSOARG00020000204	5.948620258	0.031344167	up
MSTRG.94170	6.271846698	0.01656723	up
MSTRG.219056	6.41729251	0.012438433	up
MSTRG.66010	6.441001085	0.01046453	up
MSTRG.170213	6.561767308	0.005554087	up
MSTRG.196370	6.718920484	0.039428096	up
MSTRG.3547	6.721269145	0.040696735	up
MSTRG.49841	6.765316355	0.040215083	up
MSTRG.52992	6.778512273	0.042932445	up
MSTRG.89608	6.783286178	0.036300819	up
MSTRG.141290	6.848280364	0.032406972	up
MSTRG.10859	6.864550878	0.032825437	up
MSTRG.66242	6.90931465	0.030645969	up
MSTRG.219187	6.914779909	0.031926038	up
MSTRG.258348	6.962332283	0.025762894	up
MSTRG.258351	7.055324742	0.001066332	up
ENSOARG00020019409	7.064474973	0.022365493	up
MSTRG.130216	7.072997804	0.022761266	up
MSTRG.256068	7.204899961	0.024753693	up
MSTRG.164548	7.209024379	0.017349447	up
MSTRG.230057	7.218700799	0.021023046	up
MSTRG.170780	7.296430663	0.014625563	up
MSTRG.232070	7.342442393	0.014982039	up
MSTRG.13389	7.36475755	0.000394198	up
MSTRG.56803	7.382583003	0.000159383	up
MSTRG.231992	7.387439181	0.01336185	up
MSTRG.182902	7.42239314	0.000295971	up

MSTRG.12494	7.453012207	0.009963495	up
MSTRG.70109	7.486253876	0.000616498	up
MSTRG.199719	7.681703999	0.007019713	up
MSTRG.188356	7.795649432	0.006270132	up
MSTRG.21517	7.905693564	0.005871067	up
MSTRG.161430	8.019019998	0.003987346	up
MSTRG.116886	8.210924765	0.001776765	up
MSTRG.114993	8.519409608	0.001645083	up

n white skin lncRNAs of sheep

chr	chromStart	chromEnd	name	score	strand	thickStart	thickEnd	block Count	blockSizes
X	141188945	1.41E+08	ENSOARG00020024864	0.049	-	141188945	1.41E+08	1	101,
1	16479913	16480258	ENSOARG00020000077	0.0012	+	16479913	16480258	1	345,
1	16499078	16499669	ENSOARG00020000077	0.00074	+	16499078	16499669	1	591,
1	108295164	1.08E+08	ENSOARG00020000114	4.40E-07	-	108295164	1.08E+08	1	642,
1	108294277	1.08E+08	ENSOARG00020000114	4.40E-07	-	108294277	1.08E+08	1	592,
1	108293537	1.08E+08	ENSOARG00020000114	4.40E-07	-	108293537	1.08E+08	1	396,
1	300942979	3.01E+08	ENSOARG00020000142	4.00E-07	-	300942979	3.01E+08	1	148,
1	206402658	2.06E+08	ENSOARG00020000255	0.00035	-	206402658	2.06E+08	2	462,35,
1	206379605	2.06E+08	ENSOARG00020000255	0.00019	-	206379605	2.06E+08	2	625,70,
1	206379407	2.06E+08	ENSOARG00020000255	0.00019	-	206379407	2.06E+08	1	100,
1	615182	616737	ENSOARG00020000277	0.003	+	615182	616737	2	3,397,
1	594243	595142	ENSOARG00020000277	0.0014	+	594243	595142	1	899,
1	619959	620309	ENSOARG00020000277	0.00045	+	619959	620309	1	350,
3	90940984	90941230	ENSOARG00020000327	3.20E-07	-	90940984	90941230	1	246,
3	90940641	90940740	ENSOARG00020000327	3.20E-07	-	90940641	90940740	1	99,
3	90939564	90939663	ENSOARG00020000327	3.20E-07	-	90939564	90939663	1	99,
1	231782913	2.32E+08	ENSOARG00020000428	1.90E-07	-	231782913	2.32E+08	1	200,
1	231782465	2.32E+08	ENSOARG00020000428	1.90E-07	-	231782465	2.32E+08	1	200,
4	77578155	77578305	ENSOARG00020000515	6.20E-09	+	77578155	77578305	1	150,
4	77577662	77577909	ENSOARG00020000515	6.20E-09	+	77577662	77577909	1	247,
3	193584342	1.94E+08	ENSOARG00020000519	1.90E-06	-	193584342	1.94E+08	1	198,
3	193583701	1.94E+08	ENSOARG00020000519	1.90E-06	-	193583701	1.94E+08	1	346,
3	193583110	1.94E+08	ENSOARG00020000519	1.70E-06	-	193583110	1.94E+08	1	198,
1	195374342	1.95E+08	ENSOARG00020000727	0.00023	-	195374342	1.95E+08	1	393,
3	45688789	45689437	ENSOARG00020000904	0.00056	-	45688789	45689437	1	648,
3	45687843	45688491	ENSOARG00020000904	7.60E-05	-	45687843	45688491	1	648,
1	131095310	1.31E+08	ENSOARG00020001206	8.90E-07	-	131095310	1.31E+08	1	297,
2	827961	828062	ENSOARG00020001338	0.045	-	827961	828062	1	101,
2	827167	827565	ENSOARG00020001338	0.0025	-	827167	827565	1	398,
2	836767	837265	ENSOARG00020001338	0.002	-	836767	837265	1	498,
4	107733200	1.08E+08	ENSOARG00020001359	1.00E-05	-	107733200	1.08E+08	2	54,95,
4	107730244	1.08E+08	ENSOARG00020001359	1.00E-05	-	107730244	1.08E+08	1	297,
3	63792975	63795363	ENSOARG00020001491	0.014	-	63792975	63795363	2	234,113,
3	63808766	63809656	ENSOARG00020001491	0.00089	-	63808766	63809656	1	890,
4	115530857	1.16E+08	ENSOARG00020001499	0.00021	+	115530857	1.16E+08	1	250,
1	181730014	1.82E+08	ENSOARG00020001590	0.0011	-	181730014	1.82E+08	2	145,5,
1	181729815	1.82E+08	ENSOARG00020001590	0.0011	-	181729815	1.82E+08	1	100,
1	181729466	1.82E+08	ENSOARG00020001590	0.0011	-	181729466	1.82E+08	1	200,
1	181743621	1.82E+08	ENSOARG00020001590	6.00E-05	-	181743621	1.82E+08	1	797,
2	31067535	31067785	ENSOARG00020001714	0.0011	+	31067535	31067785	1	250,
2	31067187	31067437	ENSOARG00020001714	0.0011	+	31067187	31067437	1	250,
2	31060543	31066393	ENSOARG00020001714	0.00072	+	31060543	31066393	2	25,324,
1	67318261	67318806	ENSOARG00020001988	1.60E-05	-	67318261	67318806	1	545,
3	237522711	2.38E+08	ENSOARG00020002158	8.90E-06	-	237522711	2.38E+08	1	300,
3	237521662	2.38E+08	ENSOARG00020002158	8.90E-06	-	237521662	2.38E+08	1	251,
3	237520763	2.38E+08	ENSOARG00020002158	8.90E-06	-	237520763	2.38E+08	1	151,

3	237518566	2.38E+08	ENSOARG00020002158	9.80E-07	-	237518566	2.38E+08	1	251,
1	202953608	2.03E+08	ENSOARG00020002267	0.0047	-	202953608	2.03E+08	1	249,
1	202954999	2.03E+08	ENSOARG00020002267	6.00E-04	-	202954999	2.03E+08	1	498,
3	105502477	1.06E+08	ENSOARG00020002514	2.00E-11	+	105502477	1.06E+08	1	791,
3	3189145	3190443	ENSOARG00020002667	8.90E-05	+	3189145	3190443	1	1298,
3	3191889	3192140	ENSOARG00020002667	6.30E-05	+	3191889	3192140	1	251,
3	3214325	3214725	ENSOARG00020002667	3.20E-05	+	3214325	3214725	1	400,
3	105973095	1.06E+08	ENSOARG00020002685	1.10E-06	-	105973095	1.06E+08	1	197,
2	34500391	34501576	ENSOARG00020002727	2.00E-16	+	34500391	34501576	2	696,440,
4	84118300	84118401	ENSOARG00020002769	0.0015	+	84118300	84118401	1	101,
4	84117852	84118102	ENSOARG00020002769	0.0015	+	84117852	84118102	1	250,
4	84117405	84117754	ENSOARG00020002769	0.0015	+	84117405	84117754	1	349,
4	84122180	84122579	ENSOARG00020002769	0.00049	+	84122180	84122579	1	399,
6	115957853	1.16E+08	ENSOARG00020003155	0.00014	-	115957853	1.16E+08	1	896,
14	26985622	26986070	ENSOARG00020003289	3.00E-06	+	26985622	26986070	1	448,
1	104921009	1.05E+08	ENSOARG00020003329	3.30E-05	-	104921009	1.05E+08	1	1340,
1	104920563	1.05E+08	ENSOARG00020003329	3.30E-05	-	104920563	1.05E+08	1	348,
14	7943504	7944094	ENSOARG00020003477	0.00072	-	7943504	7944094	1	590,
2	36550055	36560775	ENSOARG00020003565	0.047	-	36550055	36560775	2	151,99,
2	36320794	36321093	ENSOARG00020003565	0.00056	-	36320794	36321093	1	299,
2	36320545	36320645	ENSOARG00020003565	0.00056	-	36320545	36320645	1	100,
2	36320147	36320446	ENSOARG00020003565	0.00056	-	36320147	36320446	1	299,
2	36913703	36914003	ENSOARG00020003565	4.80E-05	-	36913703	36914003	1	300,
10	73611327	73611665	ENSOARG00020003618	1.20E-06	+	73611327	73611665	1	338,
16	10845487	10845637	ENSOARG00020003651	0.00059	+	10845487	10845637	1	150,
10	36256455	36258209	ENSOARG00020003681	0.0013	+	36256455	36258209	2	47,53,
10	36217605	36217854	ENSOARG00020003681	5.20E-05	+	36217605	36217854	1	249,
10	36203925	36204571	ENSOARG00020003681	5.20E-05	+	36203925	36204571	1	646,
10	36203231	36203778	ENSOARG00020003681	5.20E-05	+	36203231	36203778	1	547,
1	257535821	2.58E+08	ENSOARG00020003690	0.026	-	257535821	2.58E+08	3	94,65,36,
1	257526271	2.58E+08	ENSOARG00020003690	0.0011	-	257526271	2.58E+08	1	439,
1	257890536	2.58E+08	ENSOARG00020003766	0.041	+	257890536	2.58E+08	1	251,
1	257888937	2.58E+08	ENSOARG00020003766	0.015	+	257888937	2.58E+08	1	201,
1	257882995	2.58E+08	ENSOARG00020003766	0.015	+	257882995	2.58E+08	1	251,
1	257889937	2.58E+08	ENSOARG00020003766	0.0037	+	257889937	2.58E+08	1	201,
1	257889487	2.58E+08	ENSOARG00020003766	0.0037	+	257889487	2.58E+08	1	351,
1	257902063	2.58E+08	ENSOARG00020003766	0.00058	+	257902063	2.58E+08	1	201,
1	257901663	2.58E+08	ENSOARG00020003766	0.00058	+	257901663	2.58E+08	1	251,
1	257893135	2.58E+08	ENSOARG00020003766	0.00058	+	257893135	2.58E+08	3	92,898,860,
9	16962286	16962685	ENSOARG00020003863	0.0013	+	16962286	16962685	1	399,
9	16961428	16961528	ENSOARG00020003863	0.0013	+	16961428	16961528	1	100,
9	16967500	16967650	ENSOARG00020003863	0.00019	+	16967500	16967650	1	150,
9	16966952	16967351	ENSOARG00020003863	0.00019	+	16966952	16967351	1	399,
9	16938343	16938593	ENSOARG00020003863	6.60E-05	+	16938343	16938593	1	250,
9	16977430	16977778	ENSOARG00020003900	0.0048	-	16977430	16977778	2	156,92,
9	16999937	17002043	ENSOARG00020003900	0.003	-	16999937	17002043	2	310,86,
1	137886009	1.38E+08	ENSOARG00020003933	2.30E-05	+	137886009	1.38E+08	2	940,7,
1	137885362	1.38E+08	ENSOARG00020003933	2.30E-05	+	137885362	1.38E+08	1	449,

1	137927598	1.38E+08	ENSOARG00020003933	8.10E-08	+	137927598	1.38E+08	2	30,568,
13	53475138	53477508	ENSOARG00020004037	2.00E-06	+	53475138	53477508	2	1565,70,
12	53369165	53371417	ENSOARG00020004073	3.40E-05	+	53369165	53371417	3	29,48,423,
10	25600192	25600874	ENSOARG00020004322	0.00019	-	25600192	25600874	2	243,2,
7	28436500	28437539	ENSOARG00020004325	0.0031	+	28436500	28437539	2	75,169,
7	85091127	85091570	ENSOARG00020004334	4.40E-07	+	85091127	85091570	1	443,
14	68735488	68736031	ENSOARG00020004366	3.20E-12	+	68735488	68736031	1	543,
14	68734946	68735045	ENSOARG00020004366	3.20E-12	+	68734946	68735045	1	99,
8	37016191	37016392	ENSOARG00020004397	0.00023	+	37016191	37016392	1	201,
8	37133108	37133806	ENSOARG00020004408	0.00026	-	37133108	37133806	1	698,
8	37142803	37144095	ENSOARG00020004408	5.80E-05	-	37142803	37144095	3	16,285,148,
8	37702595	37703271	ENSOARG00020004553	6.60E-05	-	37702595	37703271	2	317,122,
18	5754821	5776427	ENSOARG00020004555	0.0055	+	5754821	5776427	2	61,39,
18	5785126	5785622	ENSOARG00020004555	0.001	+	5785126	5785622	1	496,
13	43273990	43275133	ENSOARG00020004700	0.00027	-	43273990	43275133	1	1143,
12	1757305	1762175	ENSOARG00020004835	1.30E-11	-	1757305	1762175	2	207,1875,
13	56855904	56856880	ENSOARG00020005298	0.0022	+	56855904	56856880	3	18,274,8,
13	56877132	56879972	ENSOARG00020005298	2.20E-05	+	56877132	56879972	1	2840,
13	56852044	56853748	ENSOARG00020005298	1.90E-05	+	56852044	56853748	2	678,220,
13	64203863	64204014	ENSOARG00020005358	5.00E-05	+	64203863	64204014	1	151,
13	64203013	64203714	ENSOARG00020005358	5.00E-05	+	64203013	64203714	1	701,
13	64202464	64202914	ENSOARG00020005358	5.00E-05	+	64202464	64202914	1	450,
13	64202164	64202365	ENSOARG00020005358	5.00E-05	+	64202164	64202365	1	201,
13	64199566	64201965	ENSOARG00020005358	5.00E-05	+	64199566	64201965	2	89,1561,
13	64199066	64199417	ENSOARG00020005358	5.00E-05	+	64199066	64199417	1	351,
10	38466154	38466604	ENSOARG00020005533	0.0049	+	38466154	38466604	1	450,
10	38465405	38465605	ENSOARG00020005533	0.0049	+	38465405	38465605	1	200,
10	38456819	38457120	ENSOARG00020005533	0.0049	+	38456819	38457120	1	301,
13	57038823	57039070	ENSOARG00020005618	3.50E-06	-	57038823	57039070	1	247,
13	57027104	57027548	ENSOARG00020005618	3.50E-06	-	57027104	57027548	1	444,
7	29802470	29921369	ENSOARG00020005761	0.00047	-	29802470	29921369	2	103,48,
7	29784726	29786615	ENSOARG00020005761	7.90E-06	-	29784726	29786615	2	51,50,
7	29782910	29784059	ENSOARG00020005761	7.90E-06	-	29782910	29784059	1	1149,
3	99563263	99563413	ENSOARG00020005773	0.037	+	99563263	99563413	1	150,
3	99609555	99609706	ENSOARG00020005773	0.00052	+	99609555	99609706	1	151,
3	99609256	99609456	ENSOARG00020005773	0.00052	+	99609256	99609456	1	200,
3	99613439	99613789	ENSOARG00020005773	8.30E-05	+	99613439	99613789	1	350,
3	99549283	99549684	ENSOARG00020005773	2.70E-05	+	99549283	99549684	1	401,
3	99548435	99549184	ENSOARG00020005773	2.70E-05	+	99548435	99549184	1	749,
8	90274659	90274809	ENSOARG00020005956	0.0013	+	90274659	90274809	1	150,
8	90268646	90269244	ENSOARG00020005956	0.00022	+	90268646	90269244	1	598,
8	90268148	90268298	ENSOARG00020005956	2.00E-04	+	90268148	90268298	1	150,
8	90267201	90267949	ENSOARG00020005956	2.00E-04	+	90267201	90267949	1	748,

6	101592368	1.02E+08	ENSOARG00020006119	4.80E-07	-	101592368	1.02E+08	1	250,
6	101590778	1.02E+08	ENSOARG00020006119	4.80E-07	-	101590778	1.02E+08	1	895,
3	164721120	1.65E+08	ENSOARG00020006202	0.00011	-	164721120	1.65E+08	3	312,495,91,
3	164756952	1.65E+08	ENSOARG00020006202	1.00E-04	-	164756952	1.65E+08	1	151,
3	164749922	1.65E+08	ENSOARG00020006202	1.00E-04	-	164749922	1.65E+08	1	749,
9	22878112	22878310	ENSOARG00020006203	3.70E-06	-	22878112	22878310	1	198,
9	22877866	22878015	ENSOARG00020006203	3.70E-06	-	22877866	22878015	1	149,
3	40599038	40600136	ENSOARG00020006215	9.80E-06	+	40599038	40600136	1	1098,
12	38357805	38358347	ENSOARG00020006251	0.0032	+	38357805	38358347	1	542,
17	75386496	75386894	ENSOARG00020006272	0.00026	-	75386496	75386894	1	398,
17	82286832	82287079	ENSOARG00020006274	9.10E-05	+	82286832	82287079	1	247,
14	69022746	69023045	ENSOARG00020006284	3.80E-06	-	69022746	69023045	1	299,
20	32811879	32820125	ENSOARG00020006450	6.00E-04	-	32811879	32820125	2	575,66,
12	70836278	70836427	ENSOARG00020006451	4.90E-05	+	70836278	70836427	1	149,
12	70835884	70836181	ENSOARG00020006451	4.90E-05	+	70835884	70836181	1	297,
12	70922270	70922517	ENSOARG00020006451	3.50E-06	+	70922270	70922517	1	247,
3	16405067	16405262	ENSOARG00020006698	1.70E-07	+	16405067	16405262	1	195,
3	16404387	16404777	ENSOARG00020006698	1.70E-07	+	16404387	16404777	1	390,
3	16403902	16404145	ENSOARG00020006698	1.70E-07	+	16403902	16404145	1	243,
2	63750813	63750963	ENSOARG00020007083	0.0014	+	63750813	63750963	1	150,
2	63749632	63749832	ENSOARG00020007083	0.0014	+	63749632	63749832	1	200,
2	63749134	63749384	ENSOARG00020007083	0.0014	+	63749134	63749384	1	250,
2	63748088	63748189	ENSOARG00020007083	0.0014	+	63748088	63748189	1	101,
2	63747640	63747840	ENSOARG00020007083	0.0014	+	63747640	63747840	1	200,
2	63747441	63747541	ENSOARG00020007083	0.0014	+	63747441	63747541	1	100,
2	63746146	63746645	ENSOARG00020007083	5.20E-05	+	63746146	63746645	1	499,
2	63745698	63745948	ENSOARG00020007083	5.20E-05	+	63745698	63745948	1	250,
2	63744353	63745052	ENSOARG00020007083	3.70E-05	+	63744353	63745052	1	699,
18	55257224	55257474	ENSOARG00020007281	0.0028	-	55257224	55257474	1	250,
18	55256776	55257076	ENSOARG00020007281	0.0028	-	55256776	55257076	1	300,
14	69594515	69600845	ENSOARG00020007353	0.0098	+	69594515	69600845	2	21,329,
14	69601842	69602492	ENSOARG00020007353	9.50E-05	+	69601842	69602492	1	650,
12	2710420	2711056	ENSOARG00020007743	1.30E-07	-	2710420	2711056	1	636,
2	39249265	39249366	ENSOARG00020007808	0.019	+	39249265	39249366	1	101,
2	39248966	39249066	ENSOARG00020007808	0.019	+	39248966	39249066	1	100,
2	39248466	39248867	ENSOARG00020007808	0.019	+	39248466	39248867	1	401,
2	39204899	39205050	ENSOARG00020007808	0.004	+	39204899	39205050	1	151,
7	22154714	22154960	ENSOARG00020007960	0.00022	+	22154714	22154960	1	246,
2	102931870	1.03E+08	ENSOARG00020007978	0.0012	-	102931870	1.03E+08	1	196,
4	52114052	52114443	ENSOARG00020008231	3.00E-07	-	52114052	52114443	1	391,
4	52113368	52113906	ENSOARG00020008231	3.00E-07	-	52113368	52113906	1	538,
7	47731583	47733097	ENSOARG00020008606	2.80E-08	+	47731583	47733097	2	124,369,
7	35255756	35256006	ENSOARG00020008835	9.10E-10	-	35255756	35256006	1	250,
4	122176708	1.22E+08	ENSOARG00020008981	5.40E-07	-	122176708	1.22E+08	1	100,
4	122176165	1.22E+08	ENSOARG00020008981	5.40E-07	-	122176165	1.22E+08	1	347,
4	122175523	1.22E+08	ENSOARG00020008981	5.40E-07	-	122175523	1.22E+08	1	149,
15	49276215	49276315	ENSOARG00020009042	0.00044	+	49276215	49276315	1	100,
15	49275468	49275618	ENSOARG00020009042	0.00044	+	49275468	49275618	1	150,
15	49275020	49275121	ENSOARG00020009042	0.00044	+	49275020	49275121	1	101,
15	49274622	49274772	ENSOARG00020009042	0.00044	+	49274622	49274772	1	150,

15	49274025	49274175	ENSOARG00020009042	0.00044	+	49274025	49274175	1	150,
15	49273079	49273827	ENSOARG00020009042	0.00044	+	49273079	49273827	1	748,
15	49272631	49272881	ENSOARG00020009042	0.00044	+	49272631	49272881	1	250,
15	49271835	49272184	ENSOARG00020009042	0.00044	+	49271835	49272184	1	349,
15	49270889	49271338	ENSOARG00020009042	0.00044	+	49270889	49271338	1	449,
15	49525482	49525881	ENSOARG00020009125	0.021	+	49525482	49525881	1	399,
15	49527923	49528073	ENSOARG00020009125	0.02	+	49527923	49528073	1	150,
15	49526478	49526628	ENSOARG00020009125	0.019	+	49526478	49526628	1	150,
15	49527126	49527625	ENSOARG00020009125	0.0041	+	49527126	49527625	1	499,
15	49524635	49525034	ENSOARG00020009125	0.0033	+	49524635	49525034	1	399,
2	156503561	1.57E+08	ENSOARG00020009200	7.10E-05	+	156503561	1.57E+08	1	100,
2	156503164	1.57E+08	ENSOARG00020009200	7.10E-05	+	156503164	1.57E+08	1	249,
2	156502568	1.57E+08	ENSOARG00020009200	7.10E-05	+	156502568	1.57E+08	1	448,
2	156498611	1.57E+08	ENSOARG00020009200	7.10E-05	+	156498611	1.57E+08	2	2,248,
2	128528136	1.29E+08	ENSOARG00020009230	4.00E-13	+	128528136	1.29E+08	2	72,420,
9	103316393	1.03E+08	ENSOARG00020009241	3.70E-06	+	103316393	1.03E+08	1	439,
10	51819479	51819579	ENSOARG00020009254	1.30E-06	-	51819479	51819579	1	100,
10	51819183	51819381	ENSOARG00020009254	1.30E-06	-	51819183	51819381	1	198,
10	32016736	32016885	ENSOARG00020009333	7.10E-07	-	32016736	32016885	1	149,
10	32016244	32016639	ENSOARG00020009333	7.10E-07	-	32016244	32016639	1	395,
10	32015899	32016097	ENSOARG00020009333	7.10E-07	-	32015899	32016097	1	198,
10	32015209	32015801	ENSOARG00020009333	7.10E-07	-	32015209	32015801	1	592,
10	32033533	32034125	ENSOARG00020009333	4.50E-07	-	32033533	32034125	1	592,
14	70786426	70786626	ENSOARG00020009680	0.03	-	70786426	70786626	1	200,
14	70787423	70787973	ENSOARG00020009680	0.015	-	70787423	70787973	1	550,
1	289394977	2.89E+08	ENSOARG00020009684	0.00015	+	289394977	2.89E+08	3	5,470,124,
1	289433622	2.89E+08	ENSOARG00020009684	1.10E-05	+	289433622	2.89E+08	2	32,667,
14	70874045	70880286	ENSOARG00020009762	0	+	70874045	70880286	3	26,96,1392,
13	69885875	69886225	ENSOARG00020009847	0.0062	-	69885875	69886225	1	350,
13	69905687	69906438	ENSOARG00020009847	0.00019	-	69905687	69906438	1	751,
13	69905438	69905539	ENSOARG00020009847	0.00019	-	69905438	69905539	1	101,
13	59564041	59564392	ENSOARG00020009985	0.0013	-	59564041	59564392	1	351,
13	59563341	59563592	ENSOARG00020009985	0.0013	-	59563341	59563592	1	251,
13	59562791	59563192	ENSOARG00020009985	0.0013	-	59562791	59563192	1	401,
13	59562242	59562542	ENSOARG00020009985	0.0013	-	59562242	59562542	1	300,
14	70959454	70962782	ENSOARG00020010054	1.50E-06	-	70959454	70962782	2	814,27,
14	70958860	70959059	ENSOARG00020010054	1.50E-06	-	70958860	70959059	1	199,
14	70958118	70958367	ENSOARG00020010054	1.50E-06	-	70958118	70958367	1	249,
14	71006884	71009868	ENSOARG00020010342	0.00017	-	71006884	71009868	2	1186,3,
19	60243830	60244231	ENSOARG00020010730	3.50E-06	-	60243830	60244231	1	401,
19	60243081	60243682	ENSOARG00020010730	3.50E-06	-	60243081	60243682	1	601,
3	89917375	89917524	ENSOARG00020010740	0.01	+	89917375	89917524	1	149,
3	89947908	89948058	ENSOARG00020010740	0.0045	+	89947908	89948058	1	150,
16	43886859	43896552	ENSOARG00020010866	8.10E-07	-	43886859	43896552	3	1275,145,580,
16	43885209	43886660	ENSOARG00020010866	8.10E-07	-	43885209	43886660	1	1451,
16	43884509	43885110	ENSOARG00020010866	8.10E-07	-	43884509	43885110	1	601,

16	43962127	44136657	ENSOARG00020010866	1.60E-08	-	43962127	44136657	3	12,594,795,
2	263682790	2.64E+08	ENSOARG00020010868	1.80E-07	-	263682790	2.64E+08	1	200,
2	263681346	2.64E+08	ENSOARG00020010868	1.80E-07	-	263681346	2.64E+08	1	200,
7	35946901	35948001	ENSOARG00020011064	0.0056	-	35946901	35948001	3	11,59,127,
7	35945760	35946707	ENSOARG00020011064	0.0056	-	35945760	35946707	3	206,43,46,
15	86750597	86750844	ENSOARG00020011092	9.80E-09	-	86750597	86750844	1	247,
15	86749876	86750401	ENSOARG00020011092	9.80E-09	-	86749876	86750401	2	393,52,
13	82000722	82001169	ENSOARG00020011453	0.027	-	82000722	82001169	1	447,
13	82010484	82010882	ENSOARG00020011453	0.012	-	82010484	82010882	1	398,
7	96964722	96964970	ENSOARG00020011478	7.10E-05	-	96964722	96964970	1	248,
7	96963631	96964078	ENSOARG00020011478	7.10E-05	-	96963631	96964078	1	447,
10	56313069	56315748	ENSOARG00020011533	6.00E-06	+	56313069	56315748	2	199,291,
8	33313173	33315013	ENSOARG00020011655	1.60E-07	-	33313173	33315013	2	52,288,
8	33308913	33309108	ENSOARG00020011655	1.60E-07	-	33308913	33309108	1	195,
6	7551010	7564239	ENSOARG00020011726	5.60E-06	-	7551010	7564239	4	791,97,77,735,
6	7551028	7551506	ENSOARG00020011726	0	-	7551028	7551506	1	478,
X	55229043	55229191	ENSOARG00020011883	0.018	-	55229043	55229191	1	148,
X	55226471	55226905	ENSOARG00020011883	0.0052	-	55226471	55226905	2	30,69,
X	55225881	55226324	ENSOARG00020011883	0.0052	-	55225881	55226324	1	443,
11	45588976	45589275	ENSOARG00020011939	0.0093	+	45588976	45589275	1	299,
11	45561607	45561856	ENSOARG00020011939	0.00087	+	45561607	45561856	1	249,
11	45560913	45561162	ENSOARG00020011939	0.00087	+	45560913	45561162	1	249,
11	45560268	45560567	ENSOARG00020011939	0.00087	+	45560268	45560567	1	299,
X	55316636	55316736	ENSOARG00020012011	0.00026	+	55316636	55316736	1	100,
X	55317373	55317620	ENSOARG00020012011	7.10E-05	+	55317373	55317620	1	247,
12	4442341	4444988	ENSOARG00020012124	8.70E-06	-	4442341	4444988	2	1437,50,
X	98615627	98615727	ENSOARG00020012139	0.0059	+	98615627	98615727	1	100,
X	98658022	98660838	ENSOARG00020012139	2.40E-07	+	98658022	98660838	2	669,76,
15	20026433	20026633	ENSOARG00020012305	0.0041	-	20026433	20026633	1	200,
15	20028667	20029165	ENSOARG00020012305	0.00035	-	20028667	20029165	1	498,
15	20029461	20030306	ENSOARG00020012305	4.00E-05	-	20029461	20030306	1	845,
15	20025143	20025590	ENSOARG00020012305	3.50E-05	-	20025143	20025590	1	447,
9	15407403	15408649	ENSOARG00020012545	2.60E-05	+	15407403	15408649	1	1246,
7	36206065	36206265	ENSOARG00020012669	0.013	+	36206065	36206265	1	200,
7	36207058	36207407	ENSOARG00020012669	0.0012	+	36207058	36207407	1	349,
7	36205221	36205520	ENSOARG00020012669	0.0011	+	36205221	36205520	1	299,
7	36204525	36204973	ENSOARG00020012669	0.0011	+	36204525	36204973	1	448,
7	36203185	36203583	ENSOARG00020012669	0.0011	+	36203185	36203583	1	398,
7	36202787	36203037	ENSOARG00020012669	0.0011	+	36202787	36203037	1	250,
7	36202390	36202540	ENSOARG00020012669	0.0011	+	36202390	36202540	1	150,
24	41472015	41472965	ENSOARG00020012785	7.80E-07	-	41472015	41472965	2	625,21,
13	76935433	76935580	ENSOARG00020013068	1.90E-07	+	76935433	76935580	1	147,
2	214255301	2.14E+08	ENSOARG00020013102	0.0034	+	214255301	2.14E+08	1	101,

2	214175366	2.14E+08	ENSOARG00020013102	0.00014	+	214175366	2.14E+08	1	600,
2	214176814	2.14E+08	ENSOARG00020013102	6.00E-05	+	214176814	2.14E+08	1	500,
2	214176465	2.14E+08	ENSOARG00020013102	6.00E-05	+	214176465	2.14E+08	1	100,
11	31035299	31035395	ENSOARG00020013346	1.90E-07	-	31035299	31035395	1	96,
11	31034916	31035108	ENSOARG00020013346	1.90E-07	-	31034916	31035108	1	192,
5	43904652	43904946	ENSOARG00020013416	3.20E-05	+	43904652	43904946	1	294,
3	108519486	1.09E+08	ENSOARG00020013451	2.00E-04	+	108519486	1.09E+08	1	1333,
3	108536342	1.09E+08	ENSOARG00020013451	0.00014	+	108536342	1.09E+08	2	7,142,
20	21735930	21737193	ENSOARG00020013553	4.10E-05	-	21735930	21737193	2	634,17,
20	21735730	21735831	ENSOARG00020013553	4.10E-05	-	21735730	21735831	1	101,
20	21742623	21746160	ENSOARG00020013553	1.00E-05	-	21742623	21746160	2	725,75,
6	75440541	75441072	ENSOARG00020013750	0.00054	+	75440541	75441072	2	445,39,
10	23077790	23078238	ENSOARG00020013869	1.00E-12	+	23077790	23078238	1	448,
10	23077443	23077593	ENSOARG00020013869	1.00E-12	+	23077443	23077593	1	150,
10	23000357	23000557	ENSOARG00020013869	1.00E-12	+	23000357	23000557	1	200,
10	23000159	23000259	ENSOARG00020013869	1.00E-12	+	23000159	23000259	1	100,
6	75905119	75917647	ENSOARG00020014026	7.80E-05	+	75905119	75917647	4	23,214,138,17,
6	75889560	75901461	ENSOARG00020014026	7.80E-05	+	75889560	75901461	2	81,18,
13	84572845	84579602	ENSOARG00020014047	0	-	84572845	84579602	3	1111,117,1770,
12	58806254	58806942	ENSOARG00020014067	2.50E-05	-	58806254	58806942	1	688,
2	265092967	2.65E+08	ENSOARG00020014108	0.00089	+	265092967	2.65E+08	1	300,
20	22409422	22409572	ENSOARG00020014136	0.049	+	22409422	22409572	1	150,
20	22394534	22403414	ENSOARG00020014136	0.049	+	22394534	22403414	3	149,42,8,
20	22393791	22394040	ENSOARG00020014136	0.00056	+	22393791	22394040	1	249,
20	22393494	22393693	ENSOARG00020014136	0.00056	+	22393494	22393693	1	199,
20	22442926	22443025	ENSOARG00020014184	0.0089	+	22442926	22443025	1	99,
20	22431403	22433704	ENSOARG00020014184	0.00059	+	22431403	22433704	2	18,817,
14	67979404	67979943	ENSOARG00020014244	0.00035	-	67979404	67979943	1	539,
16	78168662	78168909	ENSOARG00020014451	8.70E-07	-	78168662	78168909	1	247,
X	19052475	19055469	ENSOARG00020014688	2.00E-11	-	19052475	19055469	2	474,75,
X	19051357	19052006	ENSOARG00020014688	2.00E-11	-	19051357	19052006	1	649,
20	16869730	16869877	ENSOARG00020014708	3.40E-06	-	16869730	16869877	1	147,
2	216836175	2.17E+08	ENSOARG00020014748	7.90E-05	+	216836175	2.17E+08	1	201,
2	216835825	2.17E+08	ENSOARG00020014748	7.90E-05	+	216835825	2.17E+08	1	251,
2	216835525	2.17E+08	ENSOARG00020014748	7.90E-05	+	216835525	2.17E+08	1	201,
2	216835225	2.17E+08	ENSOARG00020014748	7.90E-05	+	216835225	2.17E+08	1	101,
2	216834626	2.17E+08	ENSOARG00020014748	7.90E-05	+	216834626	2.17E+08	1	300,
2	216834126	2.17E+08	ENSOARG00020014748	7.90E-05	+	216834126	2.17E+08	1	101,
2	216833726	2.17E+08	ENSOARG00020014748	7.90E-05	+	216833726	2.17E+08	1	301,
8	7356920	7357020	ENSOARG00020014763	0.018	-	7356920	7357020	1	100,
8	7351516	7351913	ENSOARG00020014763	0.018	-	7351516	7351913	1	397,
8	7316684	7316982	ENSOARG00020014763	0.018	-	7316684	7316982	1	298,
8	7316239	7316537	ENSOARG00020014763	0.018	-	7316239	7316537	1	298,
14	48398195	48398395	ENSOARG00020014795	0.0085	-	48398195	48398395	1	200,

14	48398693	48399242	ENSOARG00020014795	0.00017	-	48398693	48399242	1	549,
15	88692025	88692426	ENSOARG00020014954	0.00066	+	88692025	88692426	1	401,
1	273723675	2.74E+08	ENSOARG00020015148	0.025	+	273723675	2.74E+08	1	493,
1	273758893	2.74E+08	ENSOARG00020015148	0.013	+	273758893	2.74E+08	2	141,106,
23	2549620	2549720	ENSOARG00020015153	0.0021	+	2549620	2549720	1	100,
23	2479047	2524008	ENSOARG00020015153	0.0021	+	2479047	2524008	3	160,74,17,
23	2478848	2478949	ENSOARG00020015153	0.0021	+	2478848	2478949	1	101,
23	2549919	2550568	ENSOARG00020015153	0.00035	+	2549919	2550568	1	649,
12	82835320	82836248	ENSOARG00020015227	0.0018	-	82835320	82836248	2	100,151,
12	82832639	82833478	ENSOARG00020015227	0.0018	-	82832639	82833478	2	463,38,
12	82869268	82869869	ENSOARG00020015227	3.90E-06	-	82869268	82869869	1	601,
13	61827996	61828095	ENSOARG00020015442	3.50E-06	-	61827996	61828095	1	99,
13	61822903	61823100	ENSOARG00020015442	3.50E-06	-	61822903	61823100	1	197,
8	62614670	62615119	ENSOARG00020015455	8.10E-06	+	62614670	62615119	1	449,
1	292436678	2.92E+08	ENSOARG00020015556	0.00032	-	292436678	2.92E+08	2	7,638,
1	292278239	2.92E+08	ENSOARG00020015556	0.00013	-	292278239	2.92E+08	2	482,164,
19	60903223	60903472	ENSOARG00020015686	0.00044	+	60903223	60903472	1	249,
12	60304010	60305621	ENSOARG00020015727	2.00E-04	+	60304010	60305621	2	88,211,
15	24391999	24392194	ENSOARG00020015807	3.20E-07	+	24391999	24392194	1	195,
16	40322478	40323323	ENSOARG00020015833	3.50E-05	-	40322478	40323323	1	845,
16	40321683	40322330	ENSOARG00020015833	3.50E-05	-	40321683	40322330	1	647,
4	12168464	12168615	ENSOARG00020015965	1.60E-05	-	12168464	12168615	1	151,
4	12166268	12166419	ENSOARG00020015965	4.20E-06	-	12166268	12166419	1	151,
4	12167266	12167417	ENSOARG00020015965	1.10E-06	-	12167266	12167417	1	151,
18	57684784	57686119	ENSOARG00020016042	1.60E-05	+	57684784	57686119	1	1335,
23	2876403	2877502	ENSOARG00020016047	2.10E-05	+	2876403	2877502	1	1099,
23	2846988	2847188	ENSOARG00020016047	2.10E-05	+	2846988	2847188	1	200,
23	2846339	2846889	ENSOARG00020016047	2.10E-05	+	2846339	2846889	1	550,
23	2845890	2846041	ENSOARG00020016047	2.10E-05	+	2845890	2846041	1	151,
23	2845441	2845691	ENSOARG00020016047	2.10E-05	+	2845441	2845691	1	250,
23	2885030	2898485	ENSOARG00020016047	5.60E-06	+	2885030	2898485	3	58,68,674,
23	4174810	4175308	ENSOARG00020016223	0.011	-	4174810	4175308	1	498,
23	4174313	4174612	ENSOARG00020016223	0.011	-	4174313	4174612	1	299,
23	3820290	3821286	ENSOARG00020016223	0.011	-	3820290	3821286	1	996,
23	4176600	4177248	ENSOARG00020016223	0.0079	-	4176600	4177248	1	648,
23	4178142	4178640	ENSOARG00020016223	4.60E-06	-	4178142	4178640	1	498,
17	2331763	2332134	ENSOARG00020016287	8.70E-06	+	2331763	2332134	2	64,133,
17	2331421	2331618	ENSOARG00020016287	8.70E-06	+	2331421	2331618	1	197,
20	17589004	17589753	ENSOARG00020016306	0.0028	-	17589004	17589753	1	749,
20	17588007	17588806	ENSOARG00020016306	0.0028	-	17588007	17588806	1	799,
20	17587657	17587858	ENSOARG00020016306	0.0028	-	17587657	17587858	1	201,
20	17587458	17587559	ENSOARG00020016306	0.0028	-	17587458	17587559	1	101,
20	17587059	17587309	ENSOARG00020016306	0.0028	-	17587059	17587309	1	250,
20	17590002	17590302	ENSOARG00020016306	0.0023	-	17590002	17590302	1	300,
1	118459168	1.18E+08	ENSOARG00020016398	0.0081	-	118459168	1.18E+08	1	99,

1	118435411	1.18E+08	ENSOARG00020016398	3.40E-05	-	118435411	1.18E+08	3	340,63,37,
25	46028856	46029255	ENSOARG00020016428	0.0068	+	46028856	46029255	1	399,
14	15120298	15121945	ENSOARG00020016449	0.00019	-	15120298	15121945	1	1647,
14	15119350	15119899	ENSOARG00020016449	3.50E-05	-	15119350	15119899	1	549,
14	15122293	15125739	ENSOARG00020016449	2.60E-05	-	15122293	15125739	2	3130,15,
21	29428429	29428728	ENSOARG00020016518	0.0025	+	29428429	29428728	1	299,
12	74126877	74130571	ENSOARG00020016578	0.0048	-	74126877	74130571	2	125,75,
12	74120478	74126778	ENSOARG00020016578	0.0048	-	74120478	74126778	3	366,179,253,
12	74146272	74146821	ENSOARG00020016578	0.0039	-	74146272	74146821	1	549,
12	74145476	74146124	ENSOARG00020016578	0.0039	-	74145476	74146124	1	648,
12	74145127	74145277	ENSOARG00020016578	0.0039	-	74145127	74145277	1	150,
12	74144480	74145028	ENSOARG00020016578	0.0039	-	74144480	74145028	1	548,
12	74142336	74144381	ENSOARG00020016578	0.0039	-	74142336	74144381	2	134,664,
12	74133596	74142138	ENSOARG00020016578	0.0039	-	74133596	74142138	3	30,153,2956,
7	90024974	90025173	ENSOARG00020017106	0.002	-	90024974	90025173	1	199,
7	90048568	90048866	ENSOARG00020017106	0.0012	-	90048568	90048866	1	298,
7	90048073	90048222	ENSOARG00020017106	0.0012	-	90048073	90048222	1	149,
7	90047529	90047629	ENSOARG00020017106	5.00E-04	-	90047529	90047629	1	100,
7	90047133	90047282	ENSOARG00020017106	5.00E-04	-	90047133	90047282	1	149,
7	90045698	90046095	ENSOARG00020017106	0.00015	-	90045698	90046095	1	397,
X	73595842	73596985	ENSOARG00020017255	0.0015	+	73595842	73596985	2	9,91,
X	73436284	73436635	ENSOARG00020017255	1.40E-05	+	73436284	73436635	1	351,
X	73434536	73435636	ENSOARG00020017255	1.40E-05	+	73434536	73435636	1	1100,
X	73433686	73434137	ENSOARG00020017255	1.40E-05	+	73433686	73434137	1	451,
12	55637980	55639572	ENSOARG00020017265	1.30E-08	-	55637980	55639572	1	1592,
3	27170853	27171053	ENSOARG00020017324	2.80E-10	+	27170853	27171053	1	200,
3	27170057	27170705	ENSOARG00020017324	2.80E-10	+	27170057	27170705	1	648,
24	18771962	18772162	ENSOARG00020017350	0.00085	-	18771962	18772162	1	200,
24	18771565	18771814	ENSOARG00020017350	0.00085	-	18771565	18771814	1	249,
24	18771216	18771367	ENSOARG00020017350	0.00085	-	18771216	18771367	1	151,
24	18771017	18771118	ENSOARG00020017350	0.00085	-	18771017	18771118	1	101,
11	32827772	32827872	ENSOARG00020017351	1.60E-11	+	32827772	32827872	1	100,
11	32827129	32827625	ENSOARG00020017351	1.60E-11	+	32827129	32827625	1	496,
X	142167870	1.42E+08	ENSOARG00020017405	2.70E-09	+	142167870	1.42E+08	1	150,
X	142167571	1.42E+08	ENSOARG00020017405	2.70E-09	+	142167571	1.42E+08	1	200,
X	142167273	1.42E+08	ENSOARG00020017405	2.70E-09	+	142167273	1.42E+08	1	200,
1	119589861	1.2E+08	ENSOARG00020017884	7.20E-05	-	119589861	1.2E+08	1	100,
1	119588729	1.2E+08	ENSOARG00020017884	7.20E-05	-	119588729	1.2E+08	2	889,3,
3	4703064	4703614	ENSOARG00020018001	7.60E-06	+	4703064	4703614	1	550,
3	4702616	4702916	ENSOARG00020018001	7.60E-06	+	4702616	4702916	1	300,
3	4700821	4701520	ENSOARG00020018001	7.60E-06	+	4700821	4701520	1	699,
3	4700323	4700474	ENSOARG00020018001	7.60E-06	+	4700323	4700474	1	151,
3	4699874	4700125	ENSOARG00020018001	7.60E-06	+	4699874	4700125	1	251,
3	4744145	4744744	ENSOARG00020018001	1.20E-08	+	4744145	4744744	1	599,
3	4738543	4744046	ENSOARG00020018001	1.20E-08	+	4738543	4744046	2	66,34,
2	9702056	9702706	ENSOARG00020018041	0.00047	+	9702056	9702706	1	650,
2	9698329	9698430	ENSOARG00020018041	0.00047	+	9698329	9698430	1	101,
13	35496514	35497064	ENSOARG00020018268	7.90E-14	+	35496514	35497064	1	550,

13	35494105	35494905	ENSOARG00020018268	7.90E-14	+	35494105	35494905	1	800,
13	35493456	35493807	ENSOARG00020018268	7.90E-14	+	35493456	35493807	1	351,
26	17146114	17148556	ENSOARG00020018569	0.0089	+	17146114	17148556	2	15,234,
26	17154549	17155146	ENSOARG00020018569	4.00E-04	+	17154549	17155146	1	597,
26	17154053	17154451	ENSOARG00020018569	4.00E-04	+	17154053	17154451	1	398,
26	17153408	17153905	ENSOARG00020018569	4.00E-04	+	17153408	17153905	1	497,
6	126749409	1.27E+08	ENSOARG00020018618	7.90E-06	+	126749409	1.27E+08	1	247,
2	190695642	1.91E+08	ENSOARG00020018633	0.0083	+	190695642	1.91E+08	1	200,
2	190695092	1.91E+08	ENSOARG00020018633	0.0083	+	190695092	1.91E+08	1	401,
19	13905464	13905614	ENSOARG00020018912	0.0043	+	13905464	13905614	1	150,
19	13904915	13905365	ENSOARG00020018912	0.0043	+	13904915	13905365	1	450,
19	13904716	13904816	ENSOARG00020018912	0.0043	+	13904716	13904816	1	100,
20	32445847	32445995	ENSOARG00020019078	5.00E-12	+	32445847	32445995	1	148,
20	32444616	32445749	ENSOARG00020019078	5.00E-12	+	32444616	32445749	1	1133,
6	78901329	78901480	ENSOARG00020019405	3.00E-05	+	78901329	78901480	1	151,
6	78900532	78900732	ENSOARG00020019405	3.00E-05	+	78900532	78900732	1	200,
6	78900183	78900284	ENSOARG00020019405	3.00E-05	+	78900183	78900284	1	101,
6	78899984	78900084	ENSOARG00020019405	3.00E-05	+	78899984	78900084	1	100,
6	78899684	78899785	ENSOARG00020019405	3.00E-05	+	78899684	78899785	1	101,
6	78897934	78899586	ENSOARG00020019405	3.00E-05	+	78897934	78899586	2	42,1006,
22	45253789	45254139	ENSOARG00020019642	0.0054	+	45253789	45254139	1	350,
22	45255481	45256178	ENSOARG00020019642	0.0024	+	45255481	45256178	1	697,
22	45255083	45255283	ENSOARG00020019642	0.0024	+	45255083	45255283	1	200,
22	45258068	45258716	ENSOARG00020019642	0.00055	+	45258068	45258716	1	648,
22	45257222	45257970	ENSOARG00020019642	0.00055	+	45257222	45257970	1	748,
22	45366535	45376189	ENSOARG00020019642	3.80E-06	+	45366535	45376189	2	27,1118,
10	83614261	83614360	ENSOARG00020019655	5.80E-05	+	83614261	83614360	1	99,
10	83498489	83499029	ENSOARG00020019655	1.90E-09	+	83498489	83499029	1	540,
18	58901892	58902437	ENSOARG00020019682	0.00095	-	58901892	58902437	1	545,
3	33253659	33253807	ENSOARG00020019946	3.10E-07	-	33253659	33253807	1	148,
3	33253171	33253367	ENSOARG00020019946	3.10E-07	-	33253171	33253367	1	196,
3	33252975	33253074	ENSOARG00020019946	3.10E-07	-	33252975	33253074	1	99,
26	36110827	36128440	ENSOARG00020020026	0.021	-	36110827	36128440	2	124,27,
26	36105693	36106743	ENSOARG00020020026	0.0026	-	36105693	36106743	1	1050,
26	36108481	36108982	ENSOARG00020020026	0.00027	-	36108481	36108982	1	501,
12	49274225	49274375	ENSOARG00020020442	0.012	-	49274225	49274375	1	150,
12	49203540	49203840	ENSOARG00020020442	0.011	-	49203540	49203840	1	300,
12	49178697	49192627	ENSOARG00020020442	0.0022	-	49178697	49192627	3	424,31,44,
5	46254298	46257581	ENSOARG00020020479	9.30E-06	-	46254298	46257581	3	61,105,1520,
5	46252428	46254199	ENSOARG00020020479	9.30E-06	-	46252428	46254199	2	49,51,
25	2033675	2034123	ENSOARG00020020615	1.50E-09	+	2033675	2034123	1	448,
16	68567495	68568279	ENSOARG00020020880	1.20E-05	-	68567495	68568279	1	784,
11	35132137	35132287	ENSOARG00020021053	2.20E-07	+	35132137	35132287	1	150,
X	330211	330610	ENSOARG00020021149	0.015	-	330211	330610	1	399,
X	329664	329764	ENSOARG00020021149	0.015	-	329664	329764	1	100,
X	474466	474566	ENSOARG00020021149	1.10E-05	-	474466	474566	1	100,
X	460779	460930	ENSOARG00020021149	1.10E-05	-	460779	460930	1	151,
X	460481	460681	ENSOARG00020021149	1.10E-05	-	460481	460681	1	200,

X	460083	460333	ENSOARG00020021149	1.10E-05	-	460083	460333	1	250,
X	457993	459835	ENSOARG00020021149	1.10E-05	-	457993	459835	1	1842,
12	50500459	50500759	ENSOARG00020021178	2.60E-06	+	50500459	50500759	1	300,
12	50499760	50500360	ENSOARG00020021178	2.60E-06	+	50499760	50500360	1	600,
X	577306	577503	ENSOARG00020021235	0.0013	+	577306	577503	1	197,
12	84000921	84001120	ENSOARG00020021335	0.017	+	84000921	84001120	1	199,
2	124522173	1.25E+08	ENSOARG00020021345	0.0021	+	124522173	1.25E+08	1	699,
2	124312956	1.24E+08	ENSOARG00020021345	0.00021	+	124312956	1.24E+08	1	400,
X	725919	726017	ENSOARG00020021355	2.90E-07	+	725919	726017	1	98,
1	296796443	2.97E+08	ENSOARG00020021398	5.00E-05	+	296796443	2.97E+08	2	393,8,
3	192306555	1.92E+08	ENSOARG00020021407	1.90E-07	+	192306555	1.92E+08	1	249,
3	192306109	1.92E+08	ENSOARG00020021407	1.90E-07	+	192306109	1.92E+08	1	199,
3	192303055	1.92E+08	ENSOARG00020021407	1.90E-07	+	192303055	1.92E+08	2	79,120,
22	21794318	21794417	ENSOARG00020021489	0.042	+	21794318	21794417	1	99,
22	21793582	21793926	ENSOARG00020021489	0.0035	+	21793582	21793926	1	344,
22	21793140	21793386	ENSOARG00020021489	0.0025	+	21793140	21793386	1	246,
2	94789169	94789368	ENSOARG00020021494	3.80E-06	-	94789169	94789368	1	199,
2	94788624	94789021	ENSOARG00020021494	3.80E-06	-	94788624	94789021	1	397,
26	36511325	36511996	ENSOARG00020021500	6.60E-07	+	36511325	36511996	2	96,2,
18	21828631	21828930	ENSOARG00020021630	0.00026	+	21828631	21828930	1	299,
18	21828233	21828483	ENSOARG00020021630	0.00026	+	21828233	21828483	1	250,
X	1329941	1330734	ENSOARG00020021748	0	+	1329941	1330734	1	793,
2	164430795	1.64E+08	ENSOARG00020021805	7.20E-05	-	164430795	1.64E+08	1	300,
2	164430247	1.64E+08	ENSOARG00020021805	7.20E-05	-	164430247	1.64E+08	1	400,
X	1338574	1339072	ENSOARG00020021815	0.0054	-	1338574	1339072	1	498,
2	11404186	11404734	ENSOARG00020021825	0.018	-	11404186	11404734	1	548,
2	11372674	11373570	ENSOARG00020021825	0.0038	-	11372674	11373570	1	896,
26	21001261	21002259	ENSOARG00020021852	5.40E-05	+	21001261	21002259	1	998,
26	21000314	21000863	ENSOARG00020021852	5.40E-05	+	21000314	21000863	1	549,
26	21126257	21126457	ENSOARG00020021852	7.20E-06	+	21126257	21126457	1	200,
X	1503715	1503866	ENSOARG00020021947	0.0068	-	1503715	1503866	1	151,
X	1502717	1502968	ENSOARG00020021947	2.20E-06	-	1502717	1502968	1	251,
X	1502417	1502568	ENSOARG00020021947	2.20E-06	-	1502417	1502568	1	151,
X	1501120	1502319	ENSOARG00020021947	2.20E-06	-	1501120	1502319	1	1199,
X	1499273	1501021	ENSOARG00020021947	2.20E-06	-	1499273	1501021	1	1748,
X	1487071	1494908	ENSOARG00020021947	2.20E-06	-	1487071	1494908	2	655,893,
X	1486522	1486972	ENSOARG00020021947	2.20E-06	-	1486522	1486972	1	450,
X	1484875	1486423	ENSOARG00020021947	2.20E-06	-	1484875	1486423	1	1548,
20	11095471	11095766	ENSOARG00020022031	0.00013	+	11095471	11095766	1	295,
26	36906049	36906348	ENSOARG00020022108	0.034	+	36906049	36906348	1	299,
26	36931213	36933057	ENSOARG00020022108	1.00E-04	+	36931213	36933057	2	115,233,
24	37664759	37665258	ENSOARG00020022294	0.035	+	37664759	37665258	1	499,
24	37662919	37663020	ENSOARG00020022294	0.035	+	37662919	37663020	1	101,
24	37660772	37660972	ENSOARG00020022294	0.035	+	37660772	37660972	1	200,
24	37659548	37660623	ENSOARG00020022294	0.035	+	37659548	37660623	2	24,326,
3	224105771	2.24E+08	ENSOARG00020022300	0	+	224105771	2.24E+08	2	325,26,
3	224105421	2.24E+08	ENSOARG00020022300	0	+	224105421	2.24E+08	1	251,
12	79562049	79562300	ENSOARG00020022464	0.015	-	79562049	79562300	1	251,
12	79559900	79560150	ENSOARG00020022464	0.01	-	79559900	79560150	1	250,

12	79559400	79559651	ENSOARG00020022464	0.01	-	79559400	79559651	1	251,
12	79559100	79559301	ENSOARG00020022464	0.01	-	79559100	79559301	1	201,
12	79596222	79596323	ENSOARG00020022464	0.0014	-	79596222	79596323	1	101,
12	79595872	79596123	ENSOARG00020022464	0.0014	-	79595872	79596123	1	251,
12	79595022	79595523	ENSOARG00020022464	0.0014	-	79595022	79595523	1	501,
12	79562999	79563350	ENSOARG00020022464	2.00E-04	-	79562999	79563350	1	351,
16	8638699	8639043	ENSOARG00020022467	0.00014	+	8638699	8639043	1	344,
16	8637620	8638357	ENSOARG00020022467	0.00014	+	8637620	8638357	1	737,
4	128479298	1.28E+08	ENSOARG00020022477	0.00012	+	128479298	1.28E+08	1	896,
17	52563047	52563296	ENSOARG00020022628	0.026	-	52563047	52563296	1	249,
17	52498983	52500776	ENSOARG00020022628	0.00078	-	52498983	52500776	2	287,60<br>,
17	52498487	52498884	ENSOARG00020022628	0.00078	-	52498487	52498884	1	397,
19	15965188	15965889	ENSOARG00020022692	0.00015	+	15965188	15965889	1	701,
6	16284606	16284955	ENSOARG00020022905	4.00E-04	-	16284606	16284955	1	349,
6	16284308	16284409	ENSOARG00020022905	4.00E-04	-	16284308	16284409	1	101,
6	16281089	16284011	ENSOARG00020022905	4.00E-04	-	16281089	16284011	2	108,58<br>9,
24	34540885	34542649	ENSOARG00020022928	0.00055	-	34540885	34542649	2	169,30<br>,
24	34539991	34540588	ENSOARG00020022928	0.00055	-	34539991	34540588	1	597,
24	34539792	34539893	ENSOARG00020022928	0.00055	-	34539792	34539893	1	101,
24	34539445	34539694	ENSOARG00020022928	0.00055	-	34539445	34539694	1	249,
24	34537639	34537740	ENSOARG00020022928	0.00055	-	34537639	34537740	1	101,
22	37471726	37472026	ENSOARG00020022929	0.0022	-	37471726	37472026	1	300,
22	37468312	37468762	ENSOARG00020022929	0.0022	-	37468312	37468762	1	450,
X	6854250	6856093	ENSOARG00020022957	9.80E-05	-	6854250	6856093	1	1843,
15	27744090	27744289	ENSOARG00020023165	2.40E-07	+	27744090	27744289	1	199,
15	27743794	27743992	ENSOARG00020023165	2.40E-07	+	27743794	27743992	1	198,
15	15230421	15230865	ENSOARG00020023222	1.20E-06	+	15230421	15230865	1	444,
15	15224792	15224892	ENSOARG00020023222	1.20E-06	+	15224792	15224892	1	100,
18	31393741	31393891	ENSOARG00020023227	3.20E-05	-	31393741	31393891	1	150,
18	31392997	31393643	ENSOARG00020023227	3.20E-05	-	31392997	31393643	1	646,
18	31391756	31392898	ENSOARG00020023227	3.20E-05	-	31391756	31392898	1	1142,
18	31389254	31391658	ENSOARG00020023227	3.20E-05	-	31389254	31391658	2	357,19<br>0,
20	47824555	47824755	ENSOARG00020023310	0.03	-	47824555	47824755	1	200,
20	47824056	47824206	ENSOARG00020023310	0.01	-	47824056	47824206	1	150,
20	47823606	47823807	ENSOARG00020023310	0.01	-	47823606	47823807	1	201,
20	47829197	47829348	ENSOARG00020023310	0.0044	-	47829197	47829348	1	151,
20	47828498	47828848	ENSOARG00020023310	0.0044	-	47828498	47828848	1	350,
20	47827899	47828299	ENSOARG00020023310	0.0044	-	47827899	47828299	1	400,
20	47827050	47827700	ENSOARG00020023310	0.0044	-	47827050	47827700	1	650,
20	47826402	47826902	ENSOARG00020023310	0.0044	-	47826402	47826902	1	500,
20	47825453	47825804	ENSOARG00020023310	0.0044	-	47825453	47825804	1	351,
20	47795097	47795298	ENSOARG00020023310	0.0021	-	47795097	47795298	1	201,
20	47792400	47793349	ENSOARG00020023310	0.0021	-	47792400	47793349	1	949,
X	137613396	1.38E+08	ENSOARG00020023340	0.00038	+	137613396	1.38E+08	1	723,
7	55551012	55551302	ENSOARG00020023360	1.90E-08	-	55551012	55551302	1	290,
23	48212253	48212354	ENSOARG00020023400	0.0017	-	48212253	48212354	1	101,
23	48212054	48212154	ENSOARG00020023400	0.0017	-	48212054	48212154	1	100,
24	38551944	38553980	ENSOARG00020023451	1.40E-05	-	38551944	38553980	1	2036,
16	10492030	10492181	ENSOARG00020023457	5.60E-06	-	10492030	10492181	1	151,
16	10491731	10491832	ENSOARG00020023457	5.60E-06	-	10491731	10491832	1	101,

16	10490187	10491633	ENSOARG00020023457	5.60E-06	-	10490187	10491633	1	1446,
8	68442463	68443105	ENSOARG00020023753	2.80E-07	-	68442463	68443105	1	642,
1	214444047	2.14E+08	ENSOARG00020023804	2.10E-05	-	214444047	2.14E+08	2	459,90,
26	26721633	26722079	ENSOARG00020024174	0.00098	-	26721633	26722079	1	446,
26	26757870	26758910	ENSOARG00020024174	0.00063	-	26757870	26758910	1	1040,
26	26759057	26759305	ENSOARG00020024174	0.00049	-	26759057	26759305	1	248,
18	32499500	32499700	ENSOARG00020024281	0.025	-	32499500	32499700	1	200,
18	32478633	32479232	ENSOARG00020024281	0.0051	-	32478633	32479232	1	599,
23	48711064	48711264	ENSOARG00020024431	0.00026	+	48711064	48711264	1	200,
23	48701364	48704670	ENSOARG00020024431	3.80E-05	+	48701364	48704670	2	28,671,
26	6362425	6364301	ENSOARG00020024445	0.0025	-	6362425	6364301	2	76,24,
15	18542508	18542806	ENSOARG00020024528	0.0023	-	18542508	18542806	1	298,
21	14359840	14359990	ENSOARG00020024559	1.00E-06	-	14359840	14359990	1	150,
21	14358742	14359292	ENSOARG00020024559	1.00E-06	-	14358742	14359292	1	550,
21	14358443	14358644	ENSOARG00020024559	1.00E-06	-	14358443	14358644	1	201,
X	128232211	1.28E+08	ENSOARG00020024862	4.90E-05	-	128232211	1.28E+08	3	532,178,32,
X	141178954	1.41E+08	ENSOARG00020024864	0.0012	-	141178954	1.41E+08	1	550,
24	21970533	21970831	ENSOARG00020024892	0.00024	-	21970533	21970831	1	298,
24	21970038	21970435	ENSOARG00020024892	0.00024	-	21970038	21970435	1	397,
24	21965898	21966592	ENSOARG00020024892	0.00024	-	21965898	21966592	1	694,
19	53827656	53827957	ENSOARG00020024914	0.0034	+	53827656	53827957	1	301,
19	53827057	53827408	ENSOARG00020024914	0.0034	+	53827057	53827408	1	351,
19	53823231	53823682	ENSOARG00020024914	0.0034	+	53823231	53823682	1	451,
14	52011448	52011549	ENSOARG00020024926	0.00091	+	52011448	52011549	1	101,
14	52010750	52011349	ENSOARG00020024926	0.00091	+	52010750	52011349	1	599,
14	52012327	52013325	ENSOARG00020024926	2.60E-05	+	52012327	52013325	1	998,
23	30263046	30265089	ENSOARG00020024927	2.20E-05	-	30263046	30265089	2	277,169,
6	66779519	66779969	ENSOARG00020025295	4.80E-08	+	66779519	66779969	1	450,
9	38820842	38820943	ENSOARG00020025496	0.028	+	38820842	38820943	1	101,
9	38814987	38815486	ENSOARG00020025496	0.00071	+	38814987	38815486	1	499,
17	80682947	80683920	ENSOARG00020025543	2.20E-06	-	80682947	80683920	1	973,
3	229663933	2.3E+08	ENSOARG00020025712	0	+	229663933	2.3E+08	1	1241,
3	229892092	2.3E+08	ENSOARG00020025737	0.0024	+	229892092	2.3E+08	1	648,
3	229891744	2.3E+08	ENSOARG00020025737	0.0024	+	229891744	2.3E+08	1	101,
3	229887815	2.3E+08	ENSOARG00020025737	0.0024	+	229887815	2.3E+08	1	498,
23	67019314	67019954	ENSOARG00020025780	0.00055	+	67019314	67019954	1	640,
9	45652334	45652726	ENSOARG00020025813	0.001	-	45652334	45652726	1	392,
24	25830698	25831296	ENSOARG00020025836	5.20E-05	+	25830698	25831296	1	598,
24	25815088	25815288	ENSOARG00020025836	5.20E-05	+	25815088	25815288	1	200,
19	59384689	59395974	ENSOARG00020025886	1.30E-07	-	59384689	59395974	2	88,13,
19	59383493	59384491	ENSOARG00020025886	1.30E-07	-	59383493	59384491	1	998,
19	59383145	59383295	ENSOARG00020025886	1.30E-07	-	59383145	59383295	1	150,
19	59338278	59383046	ENSOARG00020025886	1.30E-07	-	59338278	59383046	2	57,841,
19	59942546	59942795	ENSOARG00020025953	6.00E-04	+	59942546	59942795	1	249,
19	59935909	59936702	ENSOARG00020025953	4.70E-06	+	59935909	59936702	1	793,
11	5251940	5252041	ENSOARG00020025954	2.90E-05	+	5251940	5252041	1	101,
11	5251492	5251792	ENSOARG00020025954	2.90E-05	+	5251492	5251792	1	300,
8	98242364	98242514	ENSOARG00020026186	1.30E-08	+	98242364	98242514	1	150,
8	98241619	98242216	ENSOARG00020026186	1.30E-08	+	98241619	98242216	1	597,

9	433229	433328	ENSOARG00020026208	1.10E-06	-	433229	433328	1	99,
9	432889	433133	ENSOARG00020026208	1.10E-06	-	432889	433133	1	244,
11	28346592	28347042	ENSOARG00020026217	0.0072	+	28346592	28347042	1	450,
11	28348635	28351910	ENSOARG00020026217	0.00021	+	28348635	28351910	2	674,25,
11	28345894	28346344	ENSOARG00020026217	0.00014	+	28345894	28346344	1	450,
24	2787840	2804389	ENSOARG00020026442	0.0011	+	2787840	2804389	2	620,29,
24	2780871	2782553	ENSOARG00020026442	0.00091	+	2780871	2782553	2	428,71,
24	2807964	2808563	ENSOARG00020026442	0.00078	+	2807964	2808563	1.218	599,
